# Polyunsaturated Fatty Acid Biosynthesis Involving Δ8 Desaturation and Differential DNA Methylation of *FADS2* Regulates Proliferation of Human Peripheral Blood Mononuclear Cells

**DOI:** 10.3389/fimmu.2018.00432

**Published:** 2018-03-05

**Authors:** Charlene M. Sibbons, Nicola A. Irvine, J. Eduardo Pérez-Mojica, Philip C. Calder, Karen A. Lillycrop, Barbara A. Fielding, Graham C. Burdge

**Affiliations:** ^1^Academic Unit of Human Development and Health, Faculty of Medicine, University of Southampton, Southampton, Hampshire, United Kingdom; ^2^Department of Nutritional Sciences, Faculty of Health and Medical Sciences, University of Surrey, Guildford, Surrey, United Kingdom; ^3^NIHR Southampton Biomedical Research Centre, University Hospital Southampton NHS Foundation Trust, University of Southampton, Southampton, Hampshire, United Kingdom; ^4^Centre for Biological Sciences, Faculty of Natural and Environmental Sciences, University of Southampton, Southampton, Hampshire, United Kingdom

**Keywords:** peripheral blood mononuclear cells, polyunsaturated fatty acids, desaturase, elongase, DNA methylation, cell proliferation, transcription, leukaemia

## Abstract

Polyunsaturated fatty acids (PUFAs) are important for immune function. Limited evidence indicates that immune cell activation involves endogenous PUFA synthesis, but this has not been characterised. To address this, we measured metabolism of 18:3n-3 in quiescent and activated peripheral blood mononuclear cells (PBMCs), and in Jurkat T cell leukaemia. PBMCs from men and women (*n* = 34) were incubated with [1-^13^C]18:3n-3 with or without Concanavalin A (Con. A). 18:3n-3 conversion was undetectable in unstimulated PBMCs, but up-regulated when stimulated. The main products were 20:3n-3 and 20:4n-3, while 18:4n-3 was undetectable, suggesting initial elongation and Δ8 desaturation. PUFA synthesis was 17.4-fold greater in Jurkat cells than PBMCs. The major products of 18:3n-3 conversion in Jurkat cells were 20:4n-3, 20:5n-3, and 22:5n-3. ^13^C Enrichment of 18:4n-3 and 20:3n-3 suggests parallel initial elongation and Δ6 desaturation. The FADS2 inhibitor SC26196 reduced PBMC, but not Jurkat cell, proliferation suggesting PUFA synthesis is involved in regulating mitosis in PBMCs. Con. A stimulation increased *FADS2, FADS1, ELOVL5* and *ELOVL4* mRNA expression in PBMCs. A single transcript corresponding to the major isoform of *FADS2*, FADS20001, was detected in PBMCs and Jurkat cells. PBMC activation induced hypermethylation of a 470bp region in the FADS2 5′-regulatory sequence. This region was hypomethylated in Jurkat cells compared to quiescent PBMCs. These findings show that PUFA synthesis involving initial elongation and Δ8 desaturation is involved in regulating PBMC proliferation and is regulated *via* transcription possibly by altered DNA methylation. These processes were dysregulated in Jurkat cells. This has implications for understanding the regulation of mitosis in normal and transformed lymphocytes.

## Introduction

Polyunsaturated fatty acids (PUFAs) play key roles in the immune response by acting as substrates for the synthesis of lipid second messengers involved in cell activation, including eicosanoids and for cell membrane biosynthesis (1). The omega-3 PUFA eicosapentaenoic acid (20:5n-3) and docosahexaenoic acid (22:6n-3) are substrates for the production of mediators involved in resolution of the immune response, namely resolvins, protectins and maresins (2).

**Figure 1:**
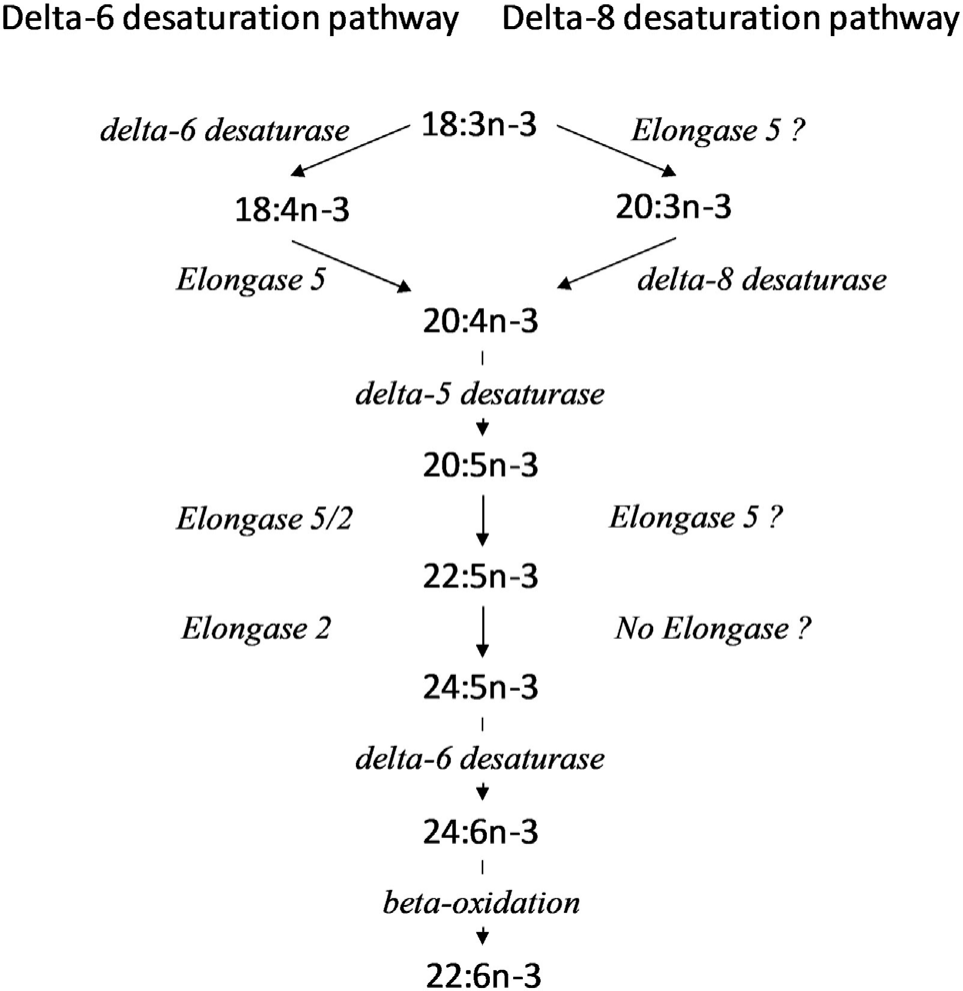
Alternative pathways for conversion of 18:3n-3 to longer chain polyunsaturated fatty acid (PUFA). ? Indicates reactions where the identity of the elongase is not known in peripheral blood mononuclear cells (PBMCs) or Jurkat cells.

Immune cells can obtain PUFA from lipoproteins and non-esterified fatty acids in blood or by synthesis from essential fatty acids. The consensus (Δ6 desaturation) pathway for conversion of α-linolenic acid (18:3n-3) to longer chain n-3 PUFA, primarily 20:5n-3, 22:5n-3, and 22:6n-3 (3–5), involves the rate limiting insertion of a double bond at the Δ6 position of 18:3n-3 which is catalysed by Δ6 desaturase (encoded by *FADS2)*, followed by chain elongation by elongase 5 (encoded by *ELOVL5*) and desaturation at the Δ5 position by Δ5 desaturase (encoded by *FADS1)*. These reactions yield 20:5n-3. Two subsequent cycles of chain elongation are catalysed by elongases 2 (encoded by *ELOVL2*) and 5, which are followed by Δ6 desaturation to yield 24:6n-3. 24:6n-3 is shortened by one cycle of peroxisomal fatty acid β-oxidation to yield 22:6n-3. However, alternative pathways have been demonstrated, although they remain poorly understood. When transfected in to yeast, the product of mammalian *FADS2* exhibits booth Δ8-desaturase and Δ6 desaturase activities, although Δ6 desaturase activity predominated (6). Initial elongation of 18:3n-3 by an as yet uncharacterised enzyme yields 20:3n-3 followed by Δ8 desaturation by the *FADS2* gene product to yield 20:4n-3 and which was subsequently converted to 20:5n-3 by Δ5 desaturation (6) (Figure 1).

There is some evidence which suggests that immune cells can convert essential fatty acids to longer chain PUFA. Incubation of murine macrophages with radiolabelled 18:2n-6 resulted in synthesis of the elongation product 20:2n-6, but no longer chain metabolites were detected (7). However, radiolabelled 18:3n-6 was converted to 20:3n-6, but not to 20:4n-6 in macrophages even when stimulated with macrophage agonists (8). Together these findings suggested that murine macrophages lack Δ6 and Δ5 desaturase activities. Activation of lymphocytes has been shown to be associated with increased synthesis of triene and tetriene PUFA, although the individual fatty acids were not identified (9). Incubation of rat lymphocytes with 18:3n-3 increased the concentrations of 20:5n-3, 22:5n-3, and 22:6n-3 (10). Treatment of human peripheral blood mononuclear cells (PBMCs) with physiological concentrations of 18:2n-6 or 18:3n-3 followed by mitogen stimulation increased the incorporation of these fatty acids into the cells (11). However, T cell receptor-stimulated splenocytes from *Fads2* null mice showed that genotype did not alter the effect dietary fatty acids on TNFα, IL-1β, IL-6, or IL-10 synthesis (12) which suggests that PUFA biosynthesis is not involved in the regulation of the production of these mediators.

T cell activation and differentiation involves changes in the mRNA expression of specific genes *via* altered epigenetic regulation, specifically DNA methylation (13). For example, differentiation of Th1 cells involves hypomethylation of the interferon-γ gene, while this gene is hypermethylated in Th2 cells (14, 15). We, and others, have shown that specific genes in the PUFA biosynthesis pathway are regulated by the DNA methylation status of their promoters (16–18). Thus, is it possible that increased PUFA biosynthesis in activated lymphocytes may involve altered epigenetic regulation of genes involved in this pathway.

Thus, although PUFA synthesis appears to be increased in activated immune cells, this process and its function have not been characterised in detail. To address this, we characterised PUFA biosynthesis in human PBMCs by determining the effect of lectin stimulation on the metabolism of [1-^13^C]18:3n-3, and on the expression and epigenetic regulation of key genes in the PUFA synthesis pathway. We also compared these findings with a spontaneously proliferating human T cell lymphoma cell line.

## Materials and Methods

### Details of Participants

Participants were healthy men and women aged 18–47 years (Table 1). Volunteers were excluded if they were pregnant, women taking hormone based contraceptives or hormone replacement therapy, had been diagnosed with type 1 or type 2 diabetes mellitus, used any prescribed medication known to affect lipid metabolism, smoked tobacco, were male and consumed more than 28 units of alcohol per week or female and consumed more than 21 units of alcohol per week, consumed more than one portion of oily fish per week, consumed fish oil supplements or were participating in another research study. After an overnight fast (at least 10 h), blood was collected from a forearm into a tube containing lithium heparin anticoagulant. PBMCs were isolated from 40 ml of heparinised blood by density gradient centrifugation using Histopaque^®^-1077 (Sigma-Aldrich, Poole, Dorset, UK) (19). Plasma was collected from the upper phase of the density gradient.

**Table 1:** Characteristics of the subjects who took part in the study.

	Men	Women	*P*
*n*	16	18	
Age (years)^a^	30 (18–38)	34 (20–47)	ns
Weight (kg)	78.8 ± 2.3	66.9 ± 2.8	<0.001
Body mass index (kg/m^2^)	24.3 ± 0.6	23.7 ± 0.7	ns
Fat mass (%)	19.3 ± 1.2	30.8 ± 1.8	<0.001
Blood metabolites			
Triacylglycerol (mmol/l)	1.2 ± 0.1	0.9 ± 0.1	ns
Total cholesterol (mmol/l)	4.5 ± 0.3	4.6 ± 0.2	ns
HDL cholesterol (mmol/l)	1.1 ± 0.1	1.7 ± 0.1	<0.0001
Fasting glucose (mmol/l)	4.9 ± 0.1	4.5 ± 0.1	ns
Haemoglobin (g/l)	14.6 ± 0.3	12.2 ± 0.3	<0.001

*Values are mean ± SEM*.

*^a^Values are mean (range)*.

### Cell Culture and Stable Isotope Labelling

Cell culture was carried out essentially as described (19). Freshly isolated PBMCs were washed with 10 ml RPMI-1640 containing 2% (v/v) autologous plasma and collected by centrifugation. For some experiments, cryopreserved PBMCs were purchased from StemCell Technologies (Vancouver, BC, Canada). Cells were resuspended in 1 ml RPMI-1640 containing 5% (v/v) autologous plasma, adjusted to 1 × 10^6^ cells/ml and maintained in a humidified incubator in an atmosphere containing 5% (v/v) CO_2_. For stable isotope labelling experiments, PBMCs (1 × 10^6^ cells/ml) were cultured in RPMI-1640 containing 5% (v/v) autologous plasma and 20 µM [1-^13^C]18:3n-3 (Campro Scientific, The Netherlands) with or without 5 µg/ml Concanavalin A (Con. A; Sigma-Aldrich). Cells were collected by centrifugation and washed as before, and then either snap frozen and stored at −80°C or were analysed immediately by flow cytometry. In some experiments, Con. A stimulated cells were treated with the Δ6 desaturase inhibitor SC26196 (200 nM) (20) or with DMSO as a vehicle control final concentration 0.02% (v/v).

The proportion of activated T lymphocytes was determined by CD69 expression (21). 5 × 10^5^ PBMCs were resuspended in 100 µl PBS. Fluorescein isothiocyanate-conjugated anti-human CD69 monoclonal antibody (Bio-Rad) (10 µl) was added and incubated for 30 min at 4°C in the dark. PBMCs were then processed for flow cytometry as described elsewhere (21).

Jurkat cells from a local archive were maintained in RPMI-1640 containing 10% (v/v) fetal bovine serum (FBS).

Cell viability was determined using either CellTiter-Glo Luminescent Cell Viability Assay (Promega) or LIVE/DEAD Fixable Red Dead Cell Stain Kit (ThermoFisher Scientific) according to the manufacturer’s instructions.

### Measurement of Cell Proliferation

Peripheral blood mononuclear cell proliferation was measured by the dye dilution method. Cryopreserved PBMCs (StemCell Technologies) were thawed and 40 × 10^6^ viable cells were suspended in 1 ml PBS containing 5% (v/v) FBS. PBMCs were either untreated or stimulated with Con. A (final concentration 5 µg/ml) and maintained in a humidified cell culture incubator at 37°C in a 5% CO_2_ atmosphere. Cells were stained with carboxyfluorescein succinimidyl ester (Affymetrix eBioscience) according to the manufacturer’s instructions.

Proliferation of Jurkat cells was measured by cell counting. Cells were seeded at 5 × 10^5^ cells/ml in RPMI-1640 medium containing 10% (v/v) FBS and incubated for up to 144 h either with or without SC26196 (200 nM) or DMSO (final concentration 0.02% (v/v)). Aliquots were collected at 24 h intervals and cell number was determined using a Coulter Z1 Cell Counter (Coulter Electronics, Luton, Essex, UK).

### Mass Spectrometry and Gas Chromatography

Cells were prepared for chromatography essentially as described (22, 23). PBMCs and Jurkat cells were collected by centrifugation, resuspended in 0.8 ml of 0.9% (w/v) NaCl and diheptadecanoylphosphatidylcholine (Sigma) internal standard (5 µg for PBMCs and 25 µg for Jurkat cells) was added to each sample. Cells were extracted with chloroform/methanol (2:1, v/v) containing 50 mg/l butylatedhydroxytoluene. 1 M NaCl (1 ml) was added, samples vortexed briefly and then centrifuged at 850 *g* for 10 min at RT. The total cell lipids extract was dried under nitrogen at 40°C and dissolved in toluene (500 µl). Methanol 2% (v/v) H_2_SO_4_ (1 ml) was added and fatty acid methyl esters (FAMEs) were synthesised by heating at 50°C for 2 h. The reaction mixture was neutralised with a solution of 0.5 M KHCO_3_ and 0.25 M K_2_CO_3_ (1 ml), and FAMEs were recovered by hexane extraction. FAMES were resolved on a J&W CP-Sil 88 fused silica capillary column (100 m × 0.25 mm × 0.2 µm) using an Agilent 6890 gas chromatograph (Agilent) equipped with a flame ionisation detection. Fatty acids were identified by their retention times relative to authentic standards.

Stable isotope enrichment was measured by gas chromatography-combustion-isotope ratio mass spectrometry as described elsewhere (24). FAMES were separated by a J&W DB-wax fused silica capillary column (30 m × 0.25 mm × 0.25 µm) (Agilent) using a Thermo Trace GC Ultra gas chromatograph (ThermoFisher) equipped with a high temperature (940°C) combustion furnace and a Thermo Finnigan Delta^Plus^ XP IRMS. The ^13^C/^12^C ratios for identified fatty acids were measured relative to a laboratory reference gas standard calibrated to the international standard Pee Dee Belemnite. Stable isotope enrichment was calculated as described (25). Total cell protein was measured using a Pierce Protein Assay kit (Thermo Fisher Scientific) according manufacturer’s instructions for a microplate procedure. The amounts of labelled fatty acids were expressed relative to the amount of cell protein.

### Quantitative RTPCR

Total RNA was extracted from cell pellets using the Qiagen RNeasy^®^ Mini kit (Qiagen) with on column DNase digestion using the RNase-free DNase set (Qiagen) according to the manufacturer’s instructions. RNA was eluted in RNase-free water (30 µl). RNA concentration was measured using a NanoDrop1000 spectrophotometer by measuring absorbance at λ 260 nm and purity was assessed using the λ 260/280 and λ 260/230 ratios. RNA integrity was checked using agarose gel electrophoresis. cDNA was synthesised by reverse transcription and qRTPCR was carried out as described (26) using commercial primers listed in (Table S1 in Supplementary Material). Transcripts were quantified using the standard curve method (27). Target transcripts were normalised using the geometric mean of four reference genes (*RPL13A, SDHA, EIF4A2*, 18s ribosomal RNA*)* which were selected for stability across treatments using the GeNorm method (28).

### Identification of FADS2 Transcripts by 5′-Rapid Amplification of cDNA Ends (5′ *RACE*)

SMARTer^®^ 5′ RACE (Clontech Laboratories) was carried out according to the manufacturer’s instructions. 1 µg of input RNA was isolated from unstimulated and stimulated PBMCs, and from Jurkat cells according to manufacturer’s instructions. Briefly, the antisense Gene specific primer for FADS2 transcript amplification was 5′GATTACGCCAAGCTTGGGCTGCCATTCGCCCAGAACAAACACG 3′, designed using Primer-BLAST software (www.ncbi.nlm.nih.gov/tools/primer-blast) and contained the sequence GATTACGCCAAGCTT to facilitate In-Fusion cloning of the PCR RACE product. RACE-ready cDNA (2.5 µl) was amplified using SeqAmp DNA polymerase (1.25 U), 2× SeqAmp buffer (25 µl), 10× UPM (5 µl), Gene Specific Primer (1 µl; final concentration of 0.2 µM), and nuclease-free water (15.5 µl) to give a total volume of 50 µl. PCR was carried out using a Veriti Thermal Cycler. PCR products were analysed using agarose gel electrophoresis and extracted from the gel using a Zymoclean gel DNA recovery kit (Zymo Research), according to manufacturer’s instructions. Gel purified products were quantified using a NanoDrop spectrophotometer and purity was assessed by agarose gel electrophoresis. SMARTer^®^ 5′ RACE products were cloned using the In-Fusion cloning kit according to the manufacturer’s instructions and transfected into Stellar Competent Cells (Clontech). Plasmid DNA was purified using a QIAprep Spin Miniprep Kit (Qiagen) according to manufacturer’s instructions and eluted in 50 µl RNase and DNase-free water. Quantity and purity was measured using a NanoDrop spectrophotometer. EcoR1 digestion was used to confirm presence of the 5′ RACE PCR product insert in the plasmid vector and the plasmids containing the RACE products were then sequenced by GATC BioTech (Germany).

### Sodium Bisulphite Pyrosequencing

We determined the effect of Con. A stimulation on the DNA methylation status of a region between −18 and −1,661 bp from the transcription start site (TSS) of *FADS2* that has been shown previously to include CpG loci that are differentially methylated in PBMCs by fatty acid supplements (29) (Figure S1 in Supplementary Material) and of a putative enhancer region (18) located in the intergenic region −7,397 bp from the *FADS2* TSS (Figure S2 in Supplementary Material). Sodium bisulphite pyrosequencing was carried out essentially as described (29). Briefly, genomic DNA was prepared and bisulphite conversion was carried out using the EZ DNA methylation kit (ZymoResearch, Irvine, CA, USA). PCR and sequencing primers are listed in Tables S2 and S3 in Supplementary Material. Modified DNA was amplified using KAPA2G Robust Hot Start Taq DNA polymerase (Labtech, Ringmer, East Sussex, UK). PCR products were immobilised on streptavidin-sepharose beads (GE Healthcare UK Ltd., Amersham, Buckinghamshire, UK), washed, denatured and released into annealing buffer containing the sequencing primers. DNA Methylation analysis was carried out using the SQA kit on a PSQ 96MA pyrosequencer (Biotage) and percentage methylation was calculated using Pyro Q CpG software (Biotage) (29).

### Statistical Analysis

Pairwise statistical comparisons were by Student’s paired or unpaired t test as appropriate. Comparisons between multiple groups were by one-way ANOVA with Tukey’s *post hoc test*. Associations were tested by linear regression. Statistical analysis was carried out using SPSS Statistics for Windows (version 24.0, IBM Corporation, Armonk, NY, USA). Sample size was calculated based on previous studies of whole body 18:3n-3 conversion. Thirty-four subjects provided 85% power to detect a 2-fold difference in synthesis of 20:5n-3 with a probability of <0.05 (30, 31). Because there were no directly comparable data of the effects of PBMC activation on the main outcomes on which to base a sample size calculation, we carried out retrospective power analysis. Twenty-six subjects provided 98% power to detect a 10% difference by Student’s paired t test in the mass of [^13^C]-labelled 20:5n-3 per unit of PBMC cell protein with α < 0.05. Six culture replicates provided 90% power to detect a 5% difference by Student’s unpaired t test in the proliferation index, division index and percent cells divided with α < 0.05. Twenty-eight subjects provided 80% power to detect a 15% difference by Student’s paired t test in *FADS2* mRNA expression with α < 0.05. Thirty-four subjects provided 89% power to detect a 5% difference by Student’s paired t test in *FADS2* DNA methylation with α < 0.05. All datasets generated for this study are included in the supplementary files. Where complete sets of measurements were not possible for technical reasons, both samples of a pair were excluded from the statistical analysis and from presentation in the results.

## Results

### Characteristics of the Subjects

Sixteen non-obese white Caucasian men and eighteen women took part in the study (Table 1). As expected, body fat mass, and HDL-cholesterol concentration were significantly higher in women compared to men. Haemoglobin concentration and weight were significantly higher in men than women (Table 1). All biochemical markers were within normal ranges.

### Cell Composition of PBMCs

There were no significant differences in the proportions of individual cells types between unstimulated and stimulated PBMCs at the end of the culture period. Unstimulated PBMCs were composed of 69.8 ± 1.7% CD3+ cells, 5.8 ± 0.5% CD19+ cells, and 3.2 ± 0.3% CD14+ cells. Stimulated PBMCs were composed of 70.5 ± 1.8% CD3+ cells, 5.6 ± 0.4% CD19+ cells, and 1.6 ± 0.3% CD14+ cells.

### Mitogen Stimulation Increases Conversion of [^13^C]18:3n-3 to Longer Chain Metabolites in PBMCs, but Is Constitutive in Jurkat Cells

**Figure 2:**
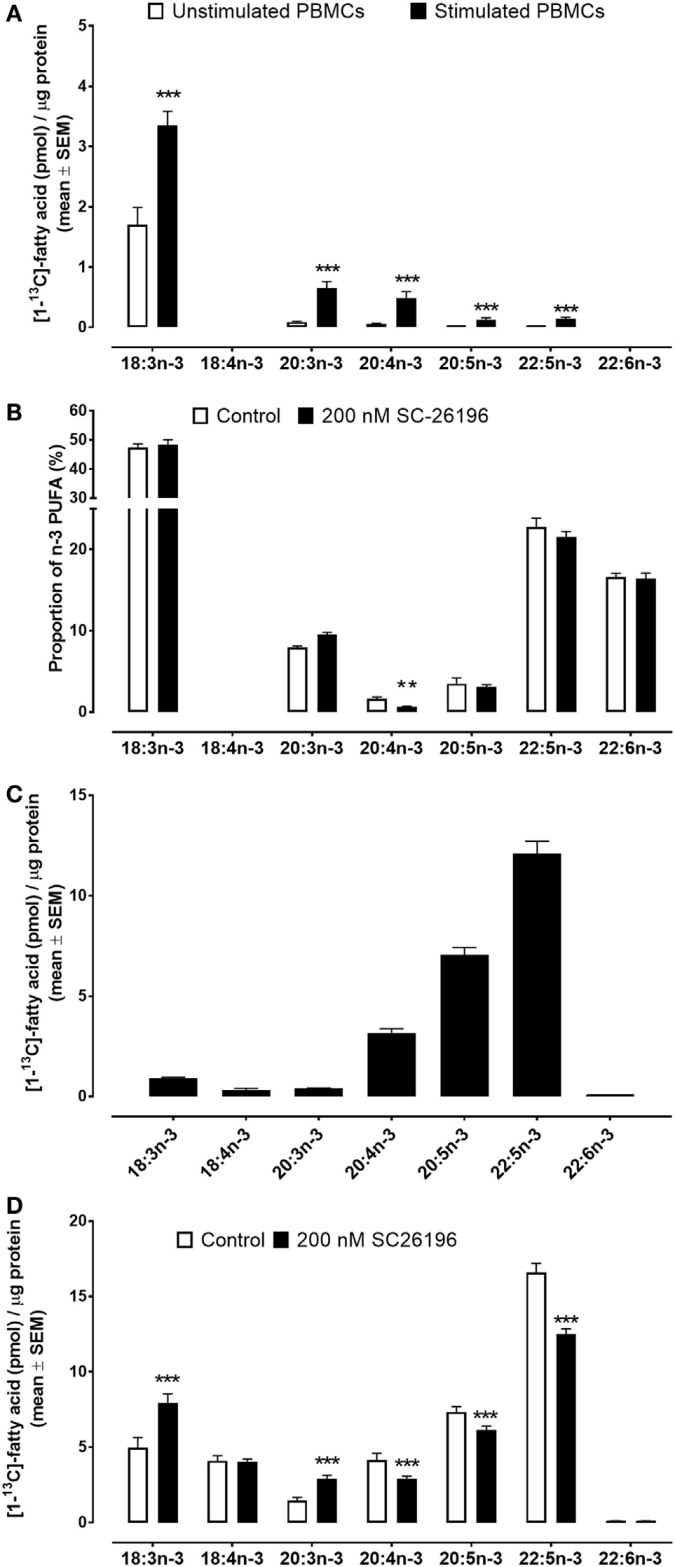
Mass of [^13^C]-labelled fatty acids per μg cell protein in peripheral blood mononuclear cells (PBMCs) and Jurkat cells incubated for 48 h with [^13^C]-18:3n-3. Values are mean ± SEM. **(A)** PBMCs from men and women (*n* = 26). **(B)** Individual n-3 polyunsaturated fatty acid (PUFA) as a proportion of total n-3 PUFA in commercially prepared PBMCs with or without SC26196 (*n* = 12 culture replicates per treatment). **(C)** [^13^C] Enrichment of n-3 PUFA in Jurkat cells (*n* = 9 culture replicates per treatment). **(D)** [^13^C]-Enrichment of n-3 PUFA in Jurkat cells with or without SC26196 (*n* = 6 culture replicates per treatment). Means that differed significantly by student’s t test are indicated by *P < 0.05, **P < 0.001, and ***P < 0.0001.

Stimulation of PBMCs with Con. A increased [^13^C] incorporation into 18:3n-3 (2.0-fold; *P* < 0.0001), 20:3n-3 (7.5-fold; *P* < 0.0001), 20:4n-3 (8.8-fold; *P* = 0.0004), 20:5n-3 (8.6-fold; *P* = 0.003), and 22:5n-3 (9.6-fold; *P* = 0.0004) compared to unstimulated PBMCs. The major products of 18:3n-3 conversion were 20:3n-3 and 20:4n-3 (Figure 2A). There was no detectable [^13^C] enrichment of either 18:4n-3 or 22:6n-3 in stimulated or unstimulated PBMCs. There was no significant difference in [^13^C] incorporation in unstimulated PBMCs between men and women (Figure S3 in Supplementary Material) and there were no significant differences between men and women in conversion of [^13^C]18:3n-3 to 20:3n-3 in stimulated PBMCs. However, synthesis of 20:5n-3 was significantly lower (68%, *P* = 0.03) in stimulated PBMCs from women compared to men (Figure S3 in Supplementary Material).

For technical reasons, [^13^C]18:3n-3 could not be used in the experiment to test the effect of SC26196 on PUFA synthesis in PBMCs. Consequently, the results are presented as the proportion of total n-3 PUFA. SC26196 significantly reduced the proportion of 20:4n-3 in stimulated PBMCs which was accompanied by a non-significant trend (*P* = 0.07) towards an increase in the proportion of 20:3n-3. There were also non-significant trends (*P* < 0.01) towards lower proportions of 20:5n-3 and 22:5n-3 (Figure 2B).

The major products of [^13^C]18:3n-3 metabolism in Jurkat leukaemia cells were 20:4n-3, 20:5n-3, and 22:5n-3 (Figure 2C). [^13^C] Enrichment was detected in both 18:4n-3 and 22:6n-3 in Jurkat cells. The amount of [^13^C]18:4n-3 synthesised over 48 h in Jurkat cells (0.32 ± 0.08 pmol/μg protein) was similar to the amount of [^13^C]20:3n-3 (0.31 ± 0.02 pmol/μg protein), but approximately 10-fold less than the amount of [^13^C]20:4n-3 (3.1 ± 0.2 pmol/μg protein). The total amount of [^13^C] PUFA synthesised from [^13^C]18:3n-3 in Jurkat cells (23.1 pmol/μg protein) was 17.4-fold greater than synthesised by stimulated PBMCs (1.3 pmol/μg protein) (*P* < 0.0001).

Treatment of Jurkat cells with SC26196 (200 nmoles/l) significantly increased [^13^C] enrichment of 18:3n-3 (59%; *P* = 0.009) and of 20:3n-3 (2-fold; *P* = 0.001), and decreased enrichment of 20:4n-3 (30%; *P* = 0.04), 20:5n-3 (19%; *P* = 0.02), and 22:5n-3 (33%; *P* = 0.0004) (Figure 2D). There was no significant effect of SC26196 on [^13^C] enrichment of 18:4n-3 and 22:6n-3.

In PBMCs, CD69 cell surface expression was associated significantly with [^13^C] enrichment of 20:3n-3 (*r* = 0.38; *P* = 0.002), 20:4n-3 (*r* = 0.33; *P* = 0.011), 20:5n-3 (*r* = 0.43; *P* = 0.0005), and 22:5n-3 (*r* = 0.45; *P* = 0.0003), but not incorporation of 18:3n-3 (Figure S4 in Supplementary Material).

### Mitogen Stimulation Increases Desaturase and Elongase mRNA Expression in PBMCs

**Figure 3:**
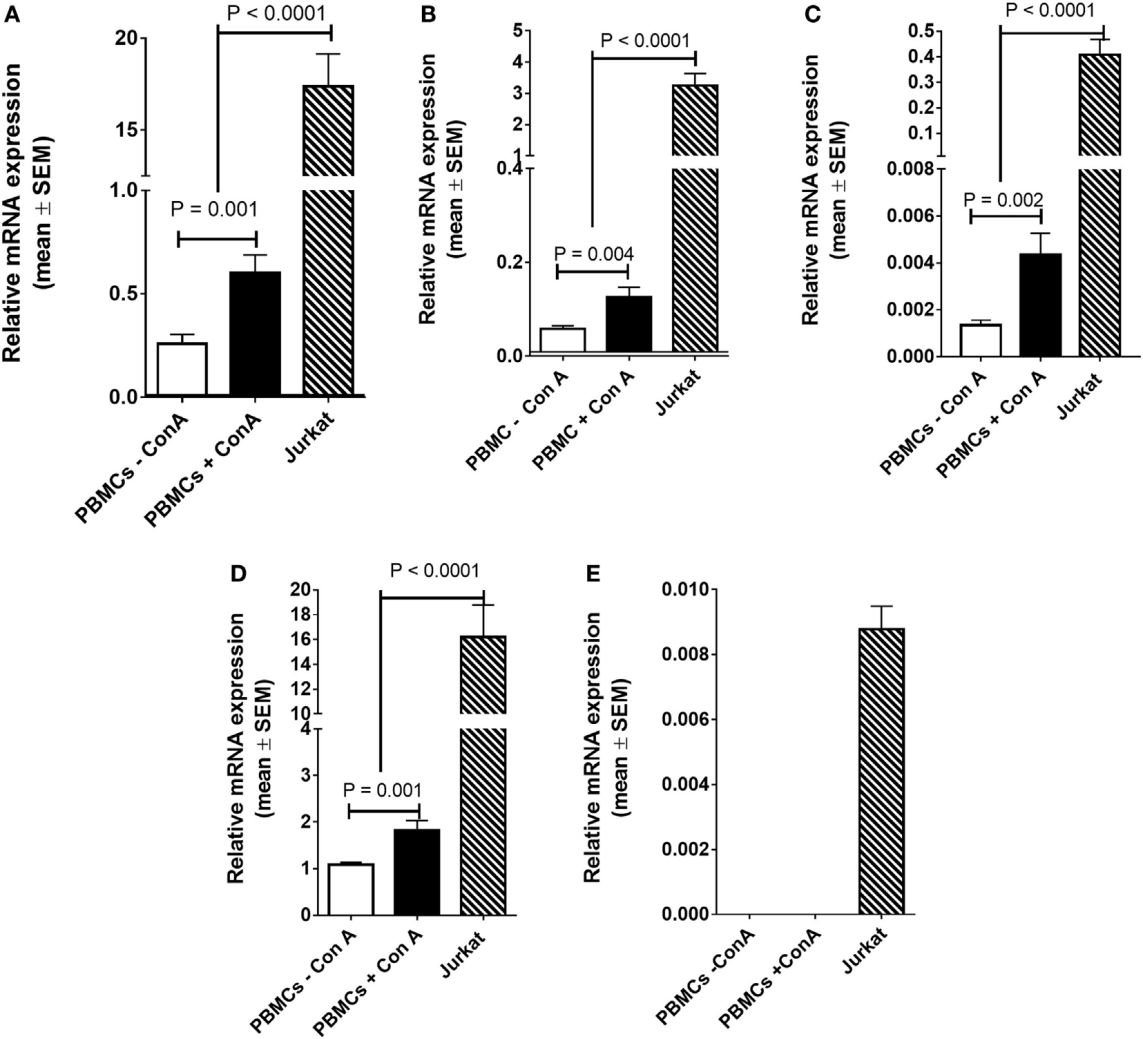
mRNA expression of **(A)** *FADS2*, **(B)** *FADS1*, **(C)** *ELOVL4*, **(D)** *ELOVL5*, and **(E)** ELOVL 2 in PBMCs either unstimulated or stimulated with Con. A from men plus women (*n* = 28), or Jurkat leukaemia cells (*n* = 10 culture replicates). Statistical analysis was by one-way ANOVA with Tukey’s *post hoc* test for multiple comparisons.

There was a significant increase (2.3-fold; *P* < 0.001) in *FADS2* mRNA expression in stimulated compared to unstimulated PBMCs (Figure 3A). Stimulation with Con. A increased the mRNA expression of *FADS1* 2.1-fold (*P* = 0.004), *ELOVL4* 3.2-fold (*P* = 0.004), and *ELOVL5* 1.6-fold (*P* = 0.001) compared to unstimulated cells (Figures 3B–D). mRNA expression of *ELOVL2* was below the limit of detection by real time RTPCR in unstimulated PBMCs. *ELOVL2* expression was detected but was too low to be quantified in 30/34 of the stimulated PBMC samples that were analysed (Figure 3E).

mRNA expression of *FADS2* was 29-fold (*P* < 0.0001), FADS1 26-fold (*P* < 0.0001), *ELOVL4* 93-fold (*P* < 0.0001), and *ELOVL5* 9-fold higher in Jurkat cells compared to stimulated PBMCs (Figures 3A–D). *ELOVL2* was expressed at a low level in Jurkat cells (Figure 3E).

### SC26196 Inhibits Proliferation of PBMCs, but Not Jurkat Cells

**Figure 4:**
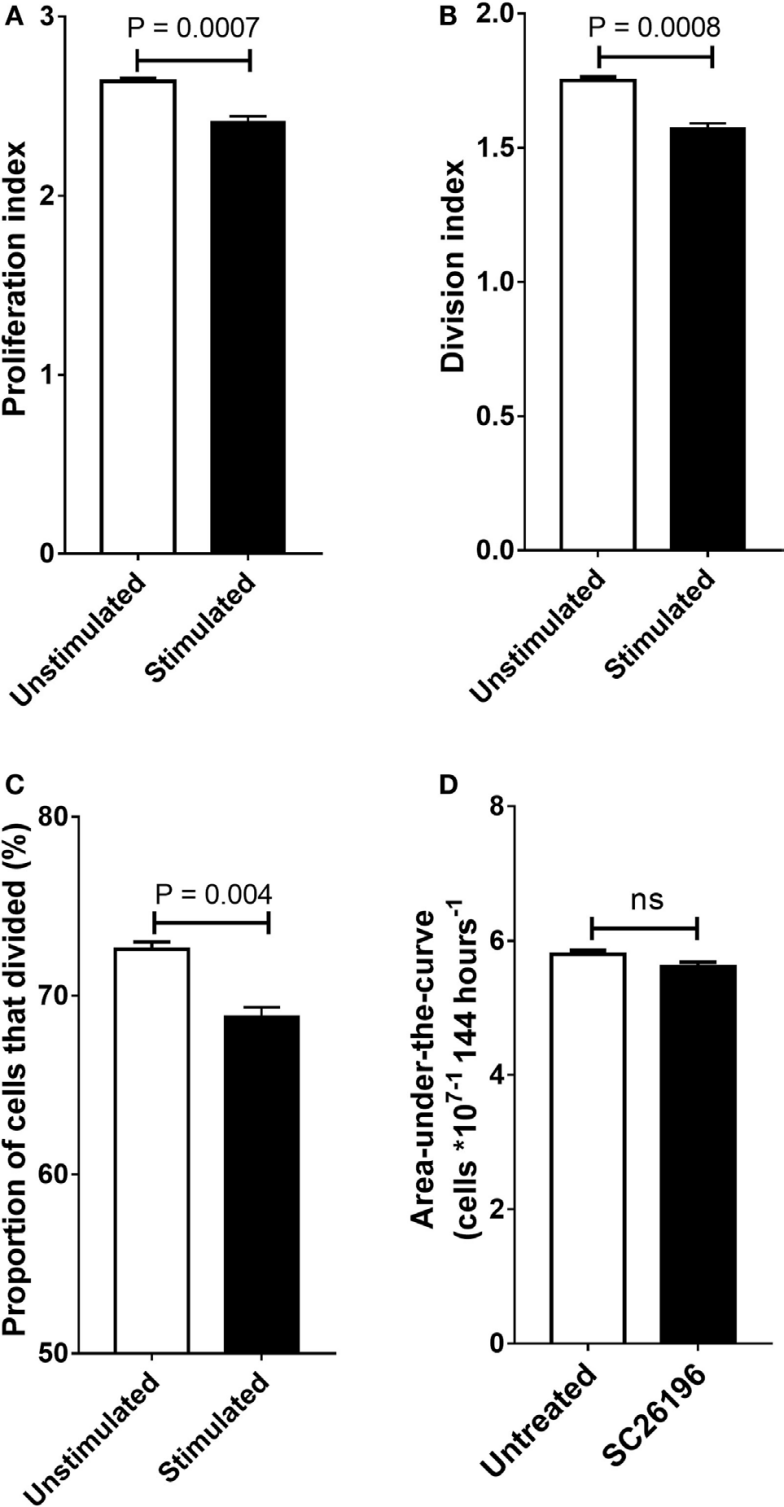
The effect of SC26196 (200 nM) on the proliferation of stimulated peripheral blood mononuclear cells (PBMCs) (*n* = 6) after 96 hours incubation **(A–C)** and on Jurkat cells (*n* = 10) **(D)**. **(A)** Proliferation index, **(B)** division index, and **(C)** proportion of cells that underwent cell division. **(D)** Jurkat cells were incubated with or without 200 nm SC26196 for 144 h. Cell proliferation was measured by cell counting. Statistical analysis of PBMCs was by Student’s paired **(A–C)** or unpaired **(D)** *t*-test.

Treatment of PBMCs with the Δ6 desaturase inhibitor SC26196 significantly decreased the proportion of cells that underwent division (Figure 4A), the division index (the mean number of cell divisions the original cell population underwent) (Figure 4B) and proliferation index (the mean number of cells divisions of the actively dividing cell population) (Figure 4C) at 96 h which corresponded to the end of the exponential growth phase. Treatment with SC26196 was associated with a small, but significant decrease in PBMC viability (2.4%; *P* = 0.003). Integration of the area-under-the cell number × time curve showed that SC26196 did not alter cell proliferation significantly in Jurkat cells (Figure 4D).

### The Same FADS2 Transcript Is Expressed in PBMCs and Jurkat Cells

**Figure 5:**
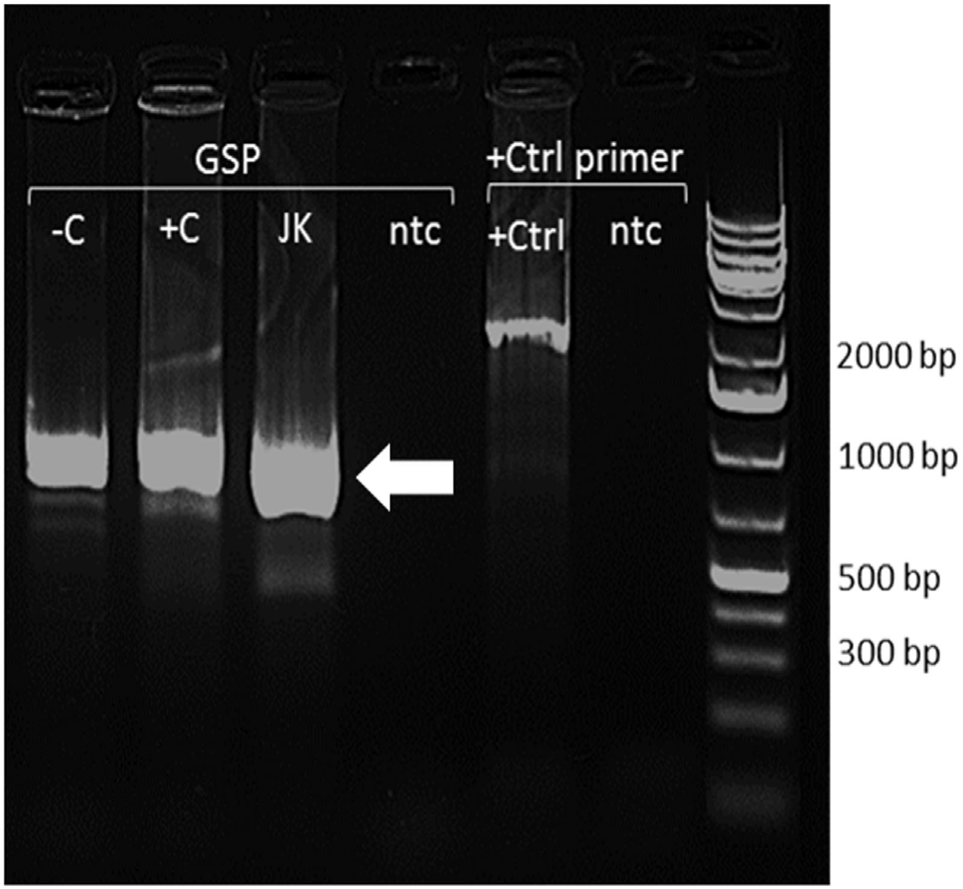
Restriction endonuclease digests of 5′ RACE PCR–pRACE clones with EcoR1 and HindIII. −C, unstimulated peripheral blood mononuclear cells (PBMCs); +C, stimulated PBMCs; JK, Jurkat cells; +Ctrl, RACE positive control; ntc, no template control. Arrow indicates *FADS2* transcript.

A single *FADS2* product of approximately 1,000 bp was detected in unstimulated and stimulated PBMCs and in Jurkat cells. EcoR1 digestion of the pRACE plasmid produced two products of approximately 400 and 600 bp. Thirteen clones were sequenced from PBMCs and 11 from Jurkat cells. Sequence analysis showed that the products matched *FADS2* variant 1 (RefSeq NM_004265.3, Ensembl transcript ID ENS 00000278840.8) and that there was no difference in the TSS between unstimulated and stimulated PBMCs, and Jurkat cells (Figure 5).

### DNA Methylation of FADS2 Is Increased in Stimulated PBMCs, but Decreased in Jurkat Cells

**Figure 6:**
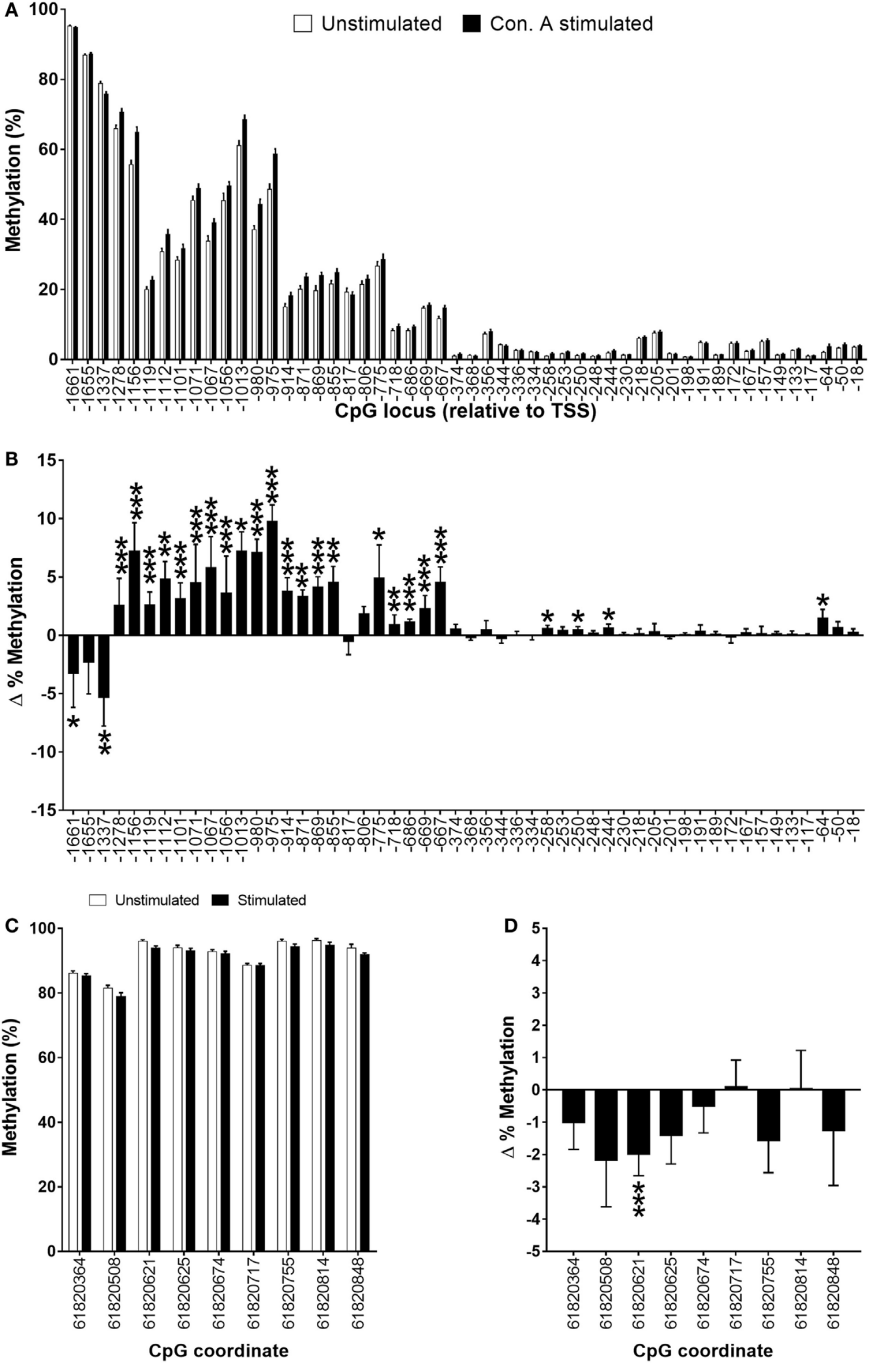
Methylation status of **(A,B)** individual CpG loci in the 5′-regulatory region of *FADS2* and **(C,D)** an intergenic putative enhancer (18) in peripheral blood mononuclear cells (PBMCs). Values are mean ± SEM, *n* = 33 paired unstimulated and Con. A stimulated PBMCs from men plus women. Means which differed significantly between stimulated and unstimulated PBMCs by Student’s paired *t*-test are indicated by *P < 0.05; **P < 0.01; ***P < 0.001. **(B,D)** show the change (stimulated minus unstimulated) in % methylation. **(A)** Methylation status of individual CpG loci in unstimulated compared to stimulated PBMCs cells. **(B)** The change in % methylation of individual CPG loci in unstimulated compared to stimulated PBMCs. **(C)** Methylation status of individual CpG loci in the 5′-regulatory region of *FADS2* in unstimulated compared to stimulated PBMCs. **(D)** The change in % methylation between of individual CpG loci in the *FADS2* putative enhancer region in unstimulated compared to stimulated PBMCs.

The methylation status of fifty-two CpG loci in a region between the TSS and 1,661 bp upstream was measured in unstimulated and stimulated PBMCs in 34 subjects. DNA methylation was highest (up to 95%) in the most distal CpG locus from the TSS, while proximal CpGs had methylation of less than 2.5% (Figure 6A). Stimulation of PBMCs decreased significantly the methylation of CpGs −1,661 (3.3%; *P* < 0.05), and −1,337 (5.3%; *P* < 0.001), and induced a non-significant decrease in CpG −1,655 compared to paired unstimulated cells (Figures 6A,B). Stimulation of PBMCs also induced a significant increase (0.9–9.8%; *P* < 0.05 to *P* < 0.001) in methylation of twenty CpG loci between −667 and 1,278 bp from the *FADS2* TSS compared to unstimulated cells (Figures 6A,B). Only two CpG loci in this region did not show a significant change in methylation. The methylation status of four further CpG loci (−258, −250, −244, −64) was also increased significantly by 0.5–1.5% (all *P* < 0.05) in stimulated compared to unstimulated PBMCs (Figures 6A,B). The methylation status of nine CpG loci was also measured in a putative enhancer region (18). The level of methylation of this region was between 79 and 95%. Stimulation of PBMCs induced decreased methylation of seven CpG loci, although only one (CpG 6182061; 2%, *P* < 0.001) reached statistical significance (Figures 6C,D). The average level of methylation across these nine CpG loci was 1% lower (*P* = 0.03) in stimulated (90.3 ± 0.3%) compared to unstimulated (91.3 ± 0.4%) PBMCs.

**Figure 7:**
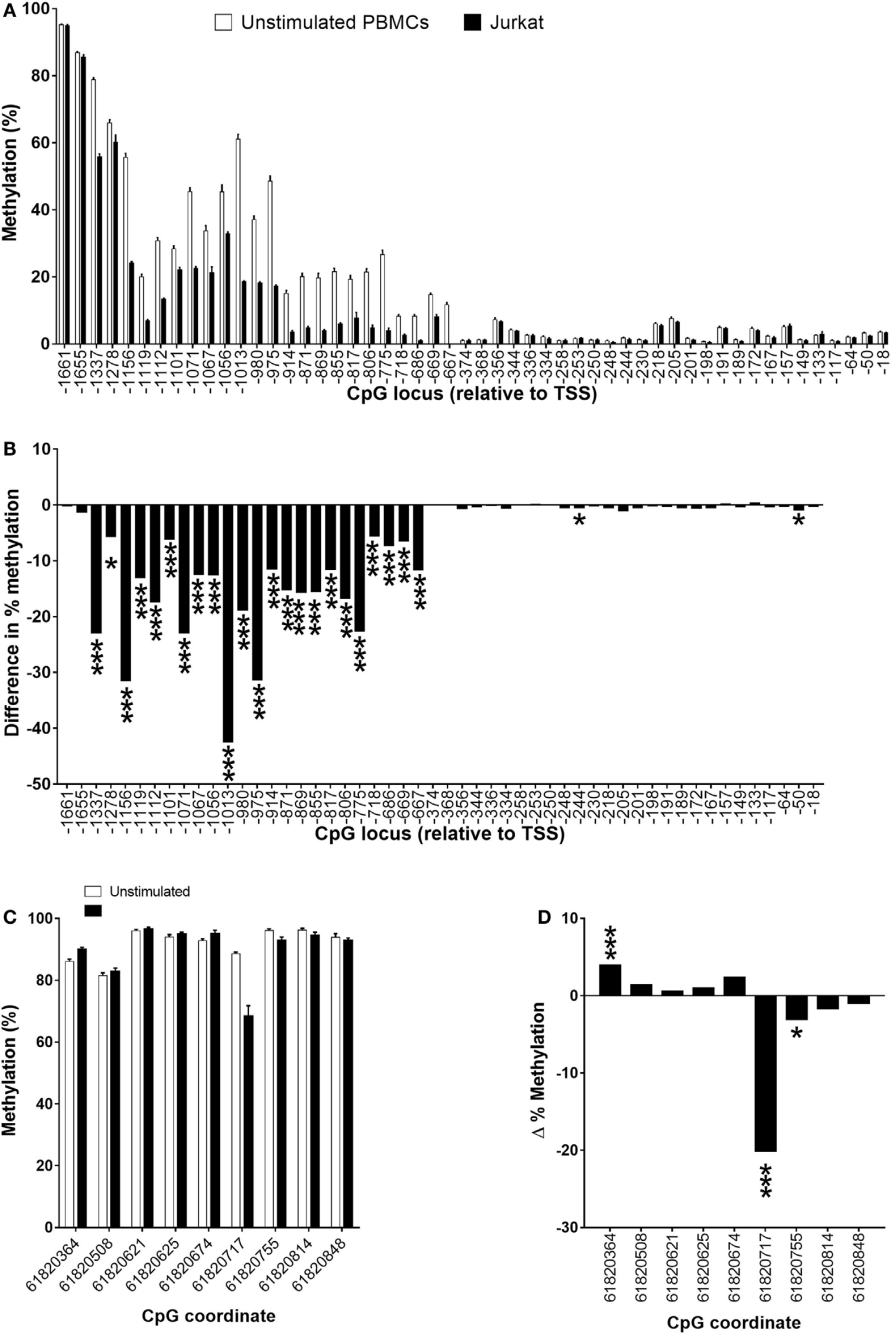
Methylation status of **(A,B)** individual CpG loci in the 5′-regulatory region of *FADS2* and **(C,D)** an intergenic putative enhancer (18) in peripheral blood mononuclear cells (PBMCs) compare to Jurkat cells. Values are mean ± SEM, *n* = 32 unstimulated PBMCs from men plus women and *n* = 10 Jurkat cell culture replicates. Means which differed significantly between stimulated and unstimulated PBMCs by Student’s paired *t*-test are indicated by *P < 0.05; **P < 0.01; ***P < 0.001. **(A)** Methylation status of individual CpG loci in unstimulated PBMCs compared to Jurkat cells. **(B)** The difference in % methylation between the mean level of methylation of individual CPG loci in PBMCs compared to Jurkat cells. **(C)** Methylation status of individual CpG loci in the 5′-regulatory region of *FADS2* in unstimulated PBMCs compared to Jurkat cells. **(D)** The difference in % methylation between the mean level of methylation of individual CpG loci in PBMCs compared to Jurkat cells. Values for unstimulated PBMCs in **(A,C)** are the same data shown in Figures 6A,C.

The level of methylation of 24 CpGs in a region between −667 and 1,655 bp relative to the TSS was significantly lower (5.6–42.5%; *P* < 0.05 to *P* < 0.001) in Jurkat cells compared to unstimulated PBMCs (Figures 7A,B). In addition, the methylation status of CpG −244 and CpG −50 was 0.5 and 0.9% lower (both *P* < 0.05) in Jurkat cells compared to unstimulated PBMCs (Figures 7A,B). DNA methylation of 3/9 CpG loci that were measured in the putative enhancer region had significantly different methylation status in Jurkat cells compared to unstimulated PBMCs (Figures 7C,D). Methylation of CpG 61820364 was significantly higher (4%; *P* < 0.001), and CpG 61820717 and CPG 61820755 had significantly lower methylation (20.1%; *P* < 0.001 and 3%; *P* < 0.05, respectively) in Jurkat cells compared to PBMCs (Figures 7C,D). In addition, four CpG loci had higher methylation and two CpG loci had lower methylation in Jurkat cells compared to unstimulated PBMCs, although these failed to reach statistical significance (Figures 7C,D). The average level of methylation across these nine CpG loci was 2% lower (*P* = 0.013) in Jurkat cells (89.3 ± 1.9%) compared to unstimulated (91.3 ± 0.4%) PBMCs.

## Discussion

The present findings show that activation of PBMCs induces increased conversion of 18:3n-3 to longer chain PUFA and that this involves increased transcription of key genes involved in this pathway and altered epigenetic regulation of *FADS2*. Enzyme activity of the PUFA biosynthesis pathway and the epigenetic regulation of *FADS2* were dysregulated in Jurkat cells. n-3 PUFA biosynthesis appears to be involved in the control of proliferation of PBMCs, but not of Jurkat cells.

The increase in conversion of essential fatty acids in stimulated cells is in agreement with the findings of Anel et al. (11), but extends these findings by identifying the individual metabolites and reactions involved. 20:3n-3 and 20:4n-3 were the major labelled metabolites of 18:3n-3 in stimulated PBMCs, while 18:4n-3 was not detected. This suggests that the first reactions of the n-3 PUFA biosynthesis pathway in PBMCs were carbon chain elongation by an unidentified elongase followed by Δ8 desaturation (Figure 1). This is in contrast to the Δ6 desaturase pathway for essential fatty acid conversion (32) (Figure 1). Despite being the major metabolite of 18:3n-3 conversion in PBMCs, 20:3n-3 accounts for only 0.05% of total lymphocyte membrane fatty acids (33). One possible interpretation is that 20:3n-3 is metabolised rapidly rather than accumulating in cell membranes. 20:3n-9 has been shown to induce anti-inflammatory effects *via* conversion to leukotriene A_3_, an inhibitor of leukotriene A_4_ hydrolase (34, 35). It is possible that 20:3n-3 may also be converted to similar immunomodulatory metabolite, although this has yet to be demonstrated.

The product of baboon *FADS2* has been shown to express both Δ6 and Δ8 desaturase activities, although predominately Δ6 desaturase, when transfected into yeast (6). However, since in the current study there was no detectable synthesis of 18:4n-3 and 22:6n-3 it appears that Δ6 desaturase activity is, at most, negligible in PBMCs. To our knowledge, this is the first demonstration of Δ8 desaturase activity in the absence of Δ6 desaturase activity in mammalian cells. Hepatic n-3 PUFA biosynthesis has been shown to be greater in women than in men (30, 31). The present findings show that conversion of 18:3n-3 to longer chain PUFA was similar in PBMCs from men and women, although synthesis of 20:5n-3 was lower in women. This suggests that sex hormones may only have a modest effect on n-3 PUFA biosynthesis in PBMCs. Treatment of PBMCs with SC26196 reduced the proportion of 20:4n-3 accompanied by a trend towards an increase in the proportion of 20:3n-3. Previous studies have shown that SC26196 inhibits Δ6 desaturase (20, 36–38). The present findings show that SC26196 can inhibit Δ8 desaturase activity in PBMCs. One possible interpretation is that SC26196 has wider specificity than reported previously and may be regarded as an inhibitor of the protein product of *FADS2* rather than of Δ6 desaturase specifically. Alternatively, the effect of SC26196 on Δ-6 desaturation may have been masked by competition between the Δ-8 and Δ-6 desaturation pathways. This may be complicated further if the desaturase activities were found to be associated with different protein isoforms.

n-3 PUFA biosynthesis was constitutive in Jurkat cells. In contrast to PBMCs, Jurkat cells expressed both Δ6 and Δ8 desaturase activities, characterised by [^13^C] enrichment of 18:4n-3, 20:3n-3, and 20:4n-3. Δ6, but not Δ8, desaturase activity has been reported previously in Jurkat cells (39). Since the amount of 18:4n-3 synthesised was approximately 3-fold greater than 20:3n-3, this suggests that Δ6 desaturation was more active than Δ8 desaturation. The major metabolites of 18:3n-3 in these cells were 20:4n-3, 20:5n-3, and 22:5n-3, which were minor metabolites in PBMCs. Furthermore, the sum of the products of 18:3n-3 conversion in Jurkat cells was approximately 17-fold greater than in PBMCs. This suggests that conversion of 18:3n-3 to longer chain metabolites in Jurkat cells is more rapid than in PBMCs. Since Jurkat cells originate from the same cell lineage as T lymphocytes, these findings imply n-3 PUFA biosynthesis is dysregulated in Jurkat cells. This may be the result of the cancer phenotype and/or adaptation to cell culture. The similarity in the amounts of 18:4n-3 and 20:3n-3 synthesised by Jurkat cells implies that the activities of the initial reactions of the Δ6 desaturase and Δ8 desaturase pathways may be comparable. If so, one possible interpretation is that simultaneous expression of both Δ8 and Δ6 activities may be due to the loss of regulatory processes that confer a single enzyme activity in T lymphocytes. SC26196 reduced Δ8 desaturation of 20:3n-3 to 20:4n-3, but not Δ6 desaturation of 18:3n-3 to 18:4n-3 in Jurkat cells. Whether this inhibitor has greater affinity for Δ8 than Δ6 desaturase activity remains to be determined.

The present findings show for the first time that up-regulation of n-3 PUFA biosynthesis is involved in, and possibly required for, mitosis in T lymphocytes. Previous reports of the effect of lymphocyte activation on n-3 PUFA biosynthesis did not investigate the role of the pathway in mitosis (11, 19). Zymosan-activated murine macrophages have been shown to convert 18:2n-6 to 20:3n-6 and, in turn, to prostaglandin (PG) E_1_. This suggests that one role of n-3 PUFA biosynthesis in activated PBMCs may be to provide substrates for the synthesis of eicosanoids that are required for proliferation. This suggestion is supported by the observation that inhibition of n-3 PUFA biosynthesis in murine aorta reduced phenylephrine-induced secretion of the proconstriction eicosanoids PGE_2_ and PGF_2α_ and partially inhibited vasoconstriction (40). In addition, polymorphisms in the *FADS1*/*FADS2* gene cluster, leading to dysregulation of n-3 PUFA biosynthesis are linked to atopic disease (41). Dietary supplementation with n-3 PUFA has been shown to reduce the activation of leukocytes and to ameliorate inflammatory disease (42). Since dietary n-3 PUFAs can down-regulate PUFA biosynthesis, the present findings suggest a novel explanation for the beneficial effects of fish oil on inflammatory disease. In contrast, inhibition of n-3 PUFA synthesis did not alter the proliferation of Jurkat cells significantly. This suggests that the link between n-3 PUFA synthesis and proliferation in T lymphocytes is disrupted in Jurkat cells which may be important in the transition from the lymphocyte to leukaemia phenotype.

We investigated whether Δ8 desaturase activity in PBMCs and Δ8/Δ6 desaturase activities in Jurkat cells were associated with expression of alternative transcripts of *FADS2. 5*′ *RACE* analysis showed that unstimulated and stimulated PBMCs, and Jurkat cells expressed a single *FADS2* transcript which corresponded to the FADS20001 isoform. This supports the findings of Park et al. which showed that the product of *FADS2* exhibits Δ8, Δ6, and Δ4 desaturase activities (6, 43), although the underlying mechanism by which different catalytic specificities are conferred simultaneously by the *FADS2* gene product is not known.

Stimulation of PBMCs increased the mRNA expression of *FADS2, FADS1*, and *ELOVL5*. Thus, activation of n-3 PUFA synthesis involves, at least in part, altered regulation of the transcription of genes that encode key enzymes in the pathway. Elongase 5 has been shown to catalyse the conversion of 18:4n-3 to 20:4n-3 in the Δ6 desaturation pathway in mammals and 18:3n-3 to 20:3n-3 in fish (44, 45). Therefore, it is possible that elongase 5 catalyses the first reaction in the Δ8 desaturation pathway in n-3 PBMCs. *ELOVL2* was not detected in unstimulated cells. *ELOVL2* expression was induced in stimulated PBMCs, but this transcript was only detected in cells from 20% of the subjects, although the level of the transcript was below the linear range of the assay. Elongase 2 catalyses conversion of 22:5n-3 to 24:5n-3 in the Δ6 desaturation pathway (32). It was not technically possible to measure conversion of 22:5n-3 to 24:5n-3 in the present study. However, it is possible that the lack of elongase 2 mRNA expression underlies 22:5n-3 being the terminal product of n-3 PUFA synthesis in PBMCs instead of 22:6n-3. This is supported by the presence of both *ELOVL2* mRNA expression and 22:6n-3 synthesis in Jurkat cells. *Elovl2* has been shown not to be expressed in murine vascular smooth muscle cells in which the PUFA synthesis pathway is required for phenylephrine-induced calcium release (40).

*ELOVL4* was upregulated in stimulated PBMCs and expressed in Jurkat cells. *ELOVL4* has only been shown previously to be expressed in retina, brain, testes and Meibomian glands (46). Hence, to our knowledge, this is the first report of the *ELOVL4* expression in the immune system. Elongase 4 catalyses the elongation of PUFA to PUFA of chain length ≥24 carbons [very long chain PUFA (VLCPUFA)]. Thus, it is unlikely that elongase 4 catalyses conversion of 18:3n-3 to 20:3n-3. However, 22:5n-3, but not 22:6n-3, has been shown to be a substrate for VLCPUFA biosynthesis in the retina (47, 48). Thus, an increase in *ELOVL4* expression leading to increased VLCPUFA synthesis would be consistent with 22:5n-3 being the terminal product of 18:3n-3 interconversion and the absence of *ELOVL2* mRNA expression. However, the marked up-regulation of *ELOVL4* in stimulated PBMCs suggest that VLCPUFA biosynthesis may play an important role in lymphocyte function. For example, sphingolipids containing C24 VLCPUFA can be involved in the formation of membrane microdomains and the associated cell signalling pathways that are involved in lymphocyte activation (49). However, to our knowledge, VLCPUFA have not been reported in PBMCs.

DNA methylation regulates the activity of specific genes during lymphocyte activation (13). We investigated the effect of stimulation of PBMCs on the DNA methylation of *FADS2* as a possible mechanism to explain the increase in the transcription of the gene. The mRNA expression of *FADS2* has been shown previously to be regulated by the DNA methylation status of specific CpG loci in the 5′ regulatory region of the gene (17, 29, 50, 51) and by a putative enhancer located in a region between the *FADS1* and *FADS2* promoters (18). Mixed cell populations may give rise to artefacts in DNA methylation analysis if the proportions of individual cell types differ between experimental groups (52). Because T lymphocytes made up approximately 70% of the PBMC preparations and there were no significant differences in the relative proportions of leukocyte populations between stimulated and unstimulated PBMCs we discounted such effects as a major influence on the DNA methylation analysis.

The findings show that CpG loci in a region between −667 and −1,661 bp relative to the TSS were differentially methylated in stimulated compared to unstimulated PBMCs. This differentially methylated region encompasses CpG loci that exhibited altered methylation status in PBMCS in response to dietary supplementation with fatty acids (29) and in HepG2 cells treated with progesterone (26). Three loci were hypomethylated, while the remainder were hypermethylated. Conventionally, increased DNA methylation is associated with suppression of transcription (53–56), although there are exceptions in which hypermethylation has been shown to promote the binding of transcription factors (57–59). Thus, it is possible that hypermethylation may increase *FADS2* transcription by preventing binding of repressive transcription factors or repressor domains. The precise effect of increased methylation of this region of FADS2 awaits requires further investigation. One CpG in the putative enhancer region was significantly hypomethylated in stimulated compared to unstimulated PBMCs and there was a small decrease in the mean methylation of this domain. The biological significance of this change cannot be deduced from these findings, although it would seem unlikely that it is functionally important.

This differentially methylated region in PBMCs, was hypomethylated in Jurkat cells compared to quiescent PBMCs. However, *FADS2* mRNA expression was higher than in stimulated PBMCs. Since increased FADS2 expression in PBMCs as associated with hypermethylation of this region, this suggests that the epigenetic regulation of *FADS2* in Jurkat cells is impaired compared to T lymphocytes. Aberrant DNA hypomethylation of genes involved in cell proliferation is an important characteristic of leukaemia cells (60). Thus, it is possible that hypomethylation of *FADS2* in Jurkat cells may have arisen as a consequence of cell transformation, although an effect of adaptation to cell culture cannot be excluded.

**Figure 8:**
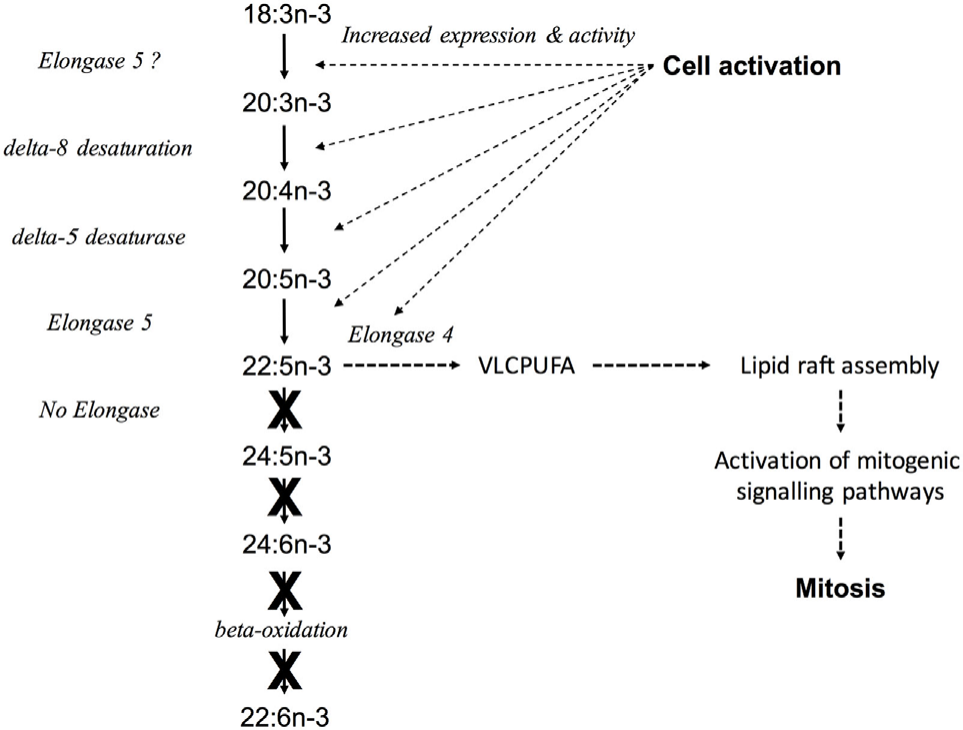
A model to explain the role of peripheral blood mononuclear cell (PUFA) synthesis in immune cell activation. Cell activation induces upregulation of the mRNA expression and activity of the enzymes encoded by *FADS1, FADS2, ELOVL5*, and *ELOVL4* leading to increased synthesis of PUFA up to 22:5n-3 *via* Δ8 desaturation. The lack of ELOVL2 mRNA expression and 22:6n-3 synthesis, accompanied by increased *ELOVL4* expression suggests that instead of interconversion to 22:6n-3, newly synthesised n-3 PUFA could be used to synthesise very long chain PUFA (VLCPUFA), which are known to be involved in the assembly of lipid rafts (49). This may facilitate the activation of signalling processes that are required for cell proliferation. Solid arrows represent known reactions, dotted arrows represent putative reactions in leukocytes. X indicates reactions that appear to not occur in leukocytes.

Together, these findings show that proliferation of PBMCs, primarily T lymphocytes, involves upregulation of n-3 PUFA synthesis by a novel pathway *via* altered the regulation of transcription key genes in the pathway, possibly involving by altered DNA methylation of *FADS2*. In addition, our findings show that proliferation of Jurkat T cell leukaemia cells is associated with dysregulation of n-3 PUFA biosynthesis that may involve changes in the regulation by DNA methylation of *FADS2*. We cautiously propose the following model to explain the role of PUFA synthesis in immune cells activation (Figure 8). Cell activation induces up-regulation of the mRNA expression and activity of the enzymes encoded by FADS2, FADS2, *ELOVL5*, and *ELOVL4*. For FADS2, at least, this involves hypermethylation of a domain in the 5′-regulatory region. The first reactions are initial chain elongation followed by Δ8 desaturation. These processes increase the synthesis of n-3 PUFA up to 22:5n-3. The apparent absence *ELOVL2* mRNA expression and of 22:6n-3 synthesis, accompanied by increased *ELOVL4* expression suggests that instead of interconversion of 18:3n-3 to 22:6n-3, newly synthesised n-3 PUFA could be used in the synthesis of VLCPUFA. Since VLCPUFA have been shown to be involved in lipid raft assembly (49), the role of PUFA synthesis in immune cells, most probably T lymphocytes, may be to provide VLCPUFA substrates that facilitate the assembly of membrane structures required for signalling processes that are responsible for inducing cell proliferation. This model does not exclude the alternative explanations for the activation of PUFA synthesis in immune cells suggested above, but suggests a mechanism that is consistent with previous findings of the role of dietary and genetic influences on immune cell function (41, 61, 62). One implication of the present data is that n-3 PUFA biosynthesis may represent a novel therapeutic target in T lymphocytes and in leukaemia cells.

## Ethics Statement

Ethical approval for the study was granted by NRES committee (REC) North West—Preston (reference 14/NW/1048) and the University of Surrey ethics committee (reference EC/2014/112/FHMS). All participants gave written informed consent.

## Author Contributions

CS, NI, and EP-M conducted the experiments and analysed the data. BF, KL, and GB designed and oversaw the study. GB wrote the first draft of the manuscript. PC contributed substantially to the final draft of the manuscript, with inputs from the other authors. All authors read and approved the manuscript.

## Conflict of Interest Statement

GB has received reimbursement for speaking at conferences sponsored by companies selling nutritional products, and are part of an academic consortium that has received research funding from Abbott Nutrition, Nestec, and Danone. He is also an advisor to BASF and a member of the BASF Asia-Pacific grant panel. PC is an advisor/consultant to Pronova BioPharma (part of BASF), DSM, Smartfish, Merck, Danone/Nutricia Research, Friesland Campina, and Fresenius-Kabi. The other authors declare no conflict of interest.
