# Supplementary material for: Polyunsaturated Fatty Acid Biosynthesis Involving Δ8 Desaturation and Differential DNA Methylation of FADS2 Regulates Proliferation of Human Peripheral Blood Mononuclear Cells
Source: Front Immunol. 2018 Mar 5;9:432. doi: 10.3389/fimmu.2018.00432 (PMC5844933; doi:10.3389/fimmu.2018.00432)
Supplement: Supplementary file 1 [file data_sheet_1.PDF]

Supplementary Material

Polyunsaturated fatty acid biosynthesis via Δ8 desaturation and differential DNA methylation of *FADS2* regulates proliferation of human peripheral blood mononuclear cells

Charlene M Sibbons, Nicola A Irvine, J Eduardo Perez-Mojica, Philip C. Calder, Karen A. Lillycrop, Margot Umpleby, Barbara A Fielding and Graham C Burdge*

*** Correspondence:** Corresponding Author: g.c.burdge@soton.ac.uk

Supplementary Table 1 Real-time RTPCR primers

Primer target	Primer Assay
FADS1	Qiagen Hs_FADS1_2_SG Quantitect Primer Assay (QT02322621)
FADS2	Qiagen Hs_FADS2_1_SG Quantitect Primer Assay (QT00077175)
ELOVL2	Qiagen Hs_ELOVL2_1_SG Quantitect Primer Assay (QT00059017)
ELOVL4	Qiagen Hs_ELOVL4_1_SG Quantitect Primer Assay (QT00017283)
ELOVL5	Qiagen Hs_ELOVL5_1_SG Quantitect Primer Assay (QT00096334)
EIF4A2	Primer design reference gene assay (HK-SY-hu)
RPL13A	Primer design reference gene assay (HK-SY-hu)
SDHA	Primer design reference gene assay (HK-SY-hu)
18S rRNA	Primer design reference gene assay (HK-SY-hu)

Supplementary Table 2 Pyrosequencing PCR primers

CpGs Covered	Forward Primer (5'-3')	Reverse Primer (5'-3')	Amplicon Size (bp)	Annealing Temperature (ºC)
Relative to FADS 2 TSS				
-1661 to -1655	GTATGGTGGTTTTGAGGATTGTT	*AAAATACTCCCTAATTCTACCTTTCAACTA	168	54
-1337 to -1156	TTGTTGTGAAATTTAGATTGGGTAGG	*CCTAAAAAAAATAAACCTAACTACAT	290	54
-1119 to -1056	*ATTTGAGGGTTTTATAATTTTTTTAGTGAT	ACCCTAATCTCAATAAACTCAATCT	319	56
-1013 to -975	GGGTAGTGATGTTGTGTAGATT	*TACACCCCACCCTCTCTAT	221	58
-914 to -855	GGTAGTTTTTATTTGTTGGAGTTTGTAT	*AAACCTCTACTCTACTTTCTTAATCT	199	56
-817 to -775	*ATTGAGTTTATTGAGATTAGGGTAAGG	ACTTTAAACCCTCTAATCAAACAATCTT	167	56
-718 to -667	ATTGTTTGATTAGAGGGTTTAAAGTT	*AAACTCCAATATCCCACATTAT	116	54
-374 to -334	GGATAATGTGGGATATTGGAGTT	*CCTACCATTAACCCAAAAAAAATCTTTC	408	60
-258 to -84	AAGATTTTTTTGGGTTAATGGT	*AAATCCCTAACTTCCCAATAC	263	56
-230 to -133	GAAAGATTTTTTTGGGTTAATGGTAG	*ATCCCTAACTTCCCAATACC	263	60
-64 to -50	*GGGGAGTTTTTATTGGAGGTAA	AATCCCTAACTTCCCAATACC	95	52
-18	TGGGGGTATTGGGAAGTTAG	*CCCTCCCCCAACCTTCTC	80	58
FADS2/FADS1 intergenic region (chromosomal location)				
61820364 to 61820508	ATGGGTTATGTTTAGTTATGTTTATTGG	*AAACAAAATCCACAACCCTATCC	231	60
61820621 to 61820755	GGTTTGGAGTTTTAGGTGGAATTTTA	*TACCTCACCCCCACAAAATAAC	232	60
61820814 to 61820848	GTTTGGAGTTTTAGGTGGAATTTTATA	*CACCCAAACTAAAATACAATAATACAAT	304	60

*Location of the biotin tag.

Supplementary Table 3 Pyrosequencing sequencing primers

CpGs Measured	Sequencing primer (5’-3’)
FADS2	
-1661, -1655	TGGTTTTGAGGATTGTTAA
-1337	AGATTGGGTAGGGTT
-1278	GGTTTTTTATTTTTAAGTGAGATG
-1156	GGGTTTTATAATTTTTTTAGTGAT
-1119, -1112, -1101	AAAATAAACCTAACTACATCC
-1071, -1067, -1056	CCTCAAACCCCAACT
-1013,	ATAGGAGGAGGGTTAG
-980, -975	GTAGATTTTAGTAAATAAAGATTGT
-914	ATTATTGTTTAATGATGTGTTTG
-871, -869, -855	TGAGATTAGGGTAAGGA
-817, -806	ACCTCTACTCTACTTTCTT
-775	CCTCTAATCAAACAATCTTAAAA
-718	AGAGGGTTTAAAGTTTTTTAAT
-686	AGGGAGGTTGTAGAAA
-669, -667	GTTAGAATGTGGATGG
-374, -368, -356, -344, -336, -334	GAGGGAGGAGGT
-258, -253, -250, -248, -244,	GATTTTTTTGGGTTAATGGTA
-230, -218, -205, -201, -198, -191, [illegible]	GGATTGGTGTAGG
-172, -167, -157, -149	GAAAGATTTTTTTGGGTTAATGGTAG
-133	GGGTAGAGGAGGTGT
-117	GAGGTTTTGAGTTTT
-84	ATTGGAGGTAAAAGTTTATAG
-64, -50	CCCAATACCCCCAAA
-18	GGGAAGTTAGGGATT
FADS2/FADS1 intergenic region	
61820364	GGAGAGAGATAAGTGAG
61820508	GGTTAGTGTTATGTATTTTGTTAT
61820621, 61820625	ATATAAATTTGTTGAATTGG
61820674	GGGAGGGTGAGGAGG
61820717	AGGAGTTTTAGATTTATTTGG
61820755	GAAATTTTGTTTTTATTAAAATATA
61820814	GGGTGAGGTAGGAGA
61820848	AAAGGTTGTAGTGAGT

```
                                                            -1661
TTATCTATATGGTATGTTAATCCATTTCTGAGAAAATAATTTTCTTGGTTCCATTTCCGT
  -1655
CAACGTTTGACAGTCCTCAAGGCCACCATACTCTCTGGAGGTCTTGCTTCATCCTCTTAT
TTTGTACAGAAGGCTTTTGTTTGGGATGTCTGCTCACACTCAAAGACAGAGAGGCCTTAC
TGTCTTTATGTCTGCAGTCTGTGGAGTGAATGAAGGATGCTCAGGGCTGACACCAGCATG
AAATGTGATGGGAGAGGTTGGGGACTGTGTGAATGTGAGGAAAGGGAGCCCATCTGCCCC
CCCAAGTACAACCCCACCAGTTGGGCAGAGTGATCCCCCCACCACCCCTGCATTGCTGTG
                      -1337
AAATTTAGATTGGGCAGGGCCCGTTTGACCATCTCTCTCAATCTCAGGCTCTCCATTTTC
                    -1278
AAGTGAGATGTAATAATATGCGTCCTGTTTACCTCTCAGCCTGTGATGAGAATCTAATGA
TTAGGGTGTGCTAGCACACAGGCACCTGTAAATCCCATTGAAATCTGAGGGCCCTATAAC
                      -1156                                -1119
TCCTCTAGTGATTCCCAAGCCTCGTGCACCCCACCCTCTCTGTTCATCCTTACTTCCCAC
      -1112           -1101                      -1071  -1067
GTGTCCCGTTAGCCCTCCGGATGCAGTCAGGCCCATTTCCCCCAGGACGCCCGGCACTAA
  -1056                                        -1013
GCCGCCCCATCCAGCTGGGGTCTGAGGGGCCTGTCTCTTGCCCCACGCCTAAAAGACCTA
                   -980    -975
ACCCTCCTCCTATCCCTTCGATACGGCAGTCTTTATTTGCTGGAGTCTGCACAACATCAC
                          -914
TGCCCAATGATGTGTCTGCTTCCACGATTCCCAAAGAGACTGAGCTTACTGAGACCAGGG
       -871-869          -855
CAAGGACCGCGCCAGTTCCTCATCGCCCCCTTCCTCTGCCTTCCCCTCCCTTCCCCCTCA
 -817       -806                           -775
CCGCAGCCATGGCGCCCAGACCAAGAAAGCAGAGCAGAGGTTCCGCAATTCTTTTCTAAG
                                         -718
ATTGTCTGACTAGAGGGTTCAAAGCCCTCTAATCCAAGGCCGGCTTGTGTCTCTAGGGAG
            -686            -669-667
GTTGCAGAAAGGCGCCAGAATGTGGATGGCGCGGACAATGTGGGATACTGGAGTCTCGAC
TGCCGCGCGCCAAGGAAGGGCGTCACCTAGCCAAGCCGGCCCTGGAGTTGAGTGCCCCGC
AGCCCGCCGGTCCACCCGCCCGGCCGCTTGCTGGCACCCTGGTGGCCGCGCCGGCTTTGT
GTCGGCATCAGGTGCAAACCCCCAGAGCGCCGGGCGTGCGCGCACGCGCCCCGGAGCGCG
CGGAGCCCATTCGTTGCCCCCACCGGGATCTCCTCTAGGATCTCCTCCGGCGCCCGGGGC
                           -374     -368         -356          -344
CGGAGAGTGGGGGAGGGAGGAGGTCGGACACGTAGCCTGCCTCGCAGCAGGGCTCGACTC
  -336-334
CACGCGGGAGGGCGGGGGAGCCGGGGACCCGCCGCTCCAGCCCGCTGGCCTTCGAAAGAT
                     -258   -253-250-248  -244               -230
CCTCCTGGGCCAATGGCAGGCGGGGCGACGCGACCGGATTGGTGCAGGCGCTCTGCTGAT
-218               -205  -201 -198   -191-189              -172   -167
CGCTGTGGAAACTCGGGCGGCGGGGAACGCGGGAGGATGTGGAACCCGAGGCGGGGGAG
 -157       -149                -133                -117
CCGGAGGGGCGGGCAGAGGAGGTGTCGAGGCCCTGAGCTCCCGGGGAGTTTTACTGGAG
                                          -64                -50
GCAAAAGTCCATAGCGGGAGGGCTGAGGGAGGGGCGGAGGAAGGGGACCGCTTGGGGGCA
                    -18
CTGGGAAGCCAGGGATCCTCCGCCAGGAAGGCAGGGACACTCCCGAGCGCAGGCGAGAAG
GCTGGGGGAGGGGGCGCGGTGGGAGGAGTAGGAGAAGACAAAAGCCGAAAGCGAAGAGGG
CCCGGGCTGCACACACCGGCTGGGAGGCAGCCGTCTGTGCAGCGAGCAGCCGGCGCGGGG
AGGCCGCAGTGCACGGGGCGTCACAGTCGGCAGGCAGCATGGGGAAGGGAGGGAACCAGG
```

Supplementary Figure 1 Location of CpG dinucleotides analysed by pyrosequencing relative to *FADS2* transcription start site (TSS). CpG dinucleotides which were measured are in bold font and their position in bp relative to the *FADS2* TSS. The underlined region is a CpG island.

```
GTGCTGGGAATACAAAGTTCAGTACTATATGGGTCATGCCTAGTCATGTCTACTGGAGGA
                61820364
GAGAGACAAGTGAGTCACGGATTATAGGTCAGTAGGATAGATGCAAATTAAATTCATCTT

ACTCAGGATTTAAACAGCTTATATGACATGACTTTGCATTCATGTTTCTTTGCTTTTCAT
                                          61820508
GTGTTCTTAGGTCAGTGTCATGTATTTTGCTACCTCCCTAACGAGCTTATAATCCAGGAT

AGGGCTGTGGATTCTGTTTATACTACCAATACATATTCAGAGCACCTTGGTTTGGAGTTC
                                  61820621 61820625
TAGGTGGAATCCCACATAAATTTGCTGAATTGGCCGGGCGTGGCAGCTCATGCCTGTAAT
                            61820674
CCCTGCACTTTGGGAGGGTGAGGAGGGCGGATCACCTGAGGTCAGGAGCTCCAGACCCAC
        61820717                                    61820755
TTGGCCAACACGGTGAAACCCTGTCTCTACTAAAATACAAAAATTAGCCGGGTGTGGTGG
                                                 61820814
GGGGTGCCTGTAATCCCAGCTACTTTGTGGGGGTGAGGCAGGAGAATCGCTTGAACCCAG
                    61820848
GAGGCAAAGGTTGCAGTGAGCCGAGATTGCACCACTGCACTCCAGCCTGGGTGACAGAGC
```

Supplementary Figure 2 Location of CpG dinucleotides in the *FADS2/FADS1* intergenic region that were analysed by pyrosequencing. The CpG that were measured are in bold font and with the chromosomal coordinate (assembly GRCh38).

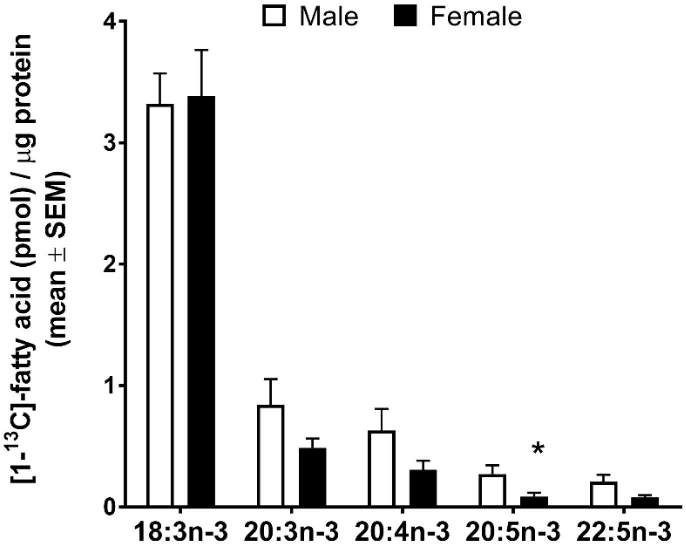

Supplementary Figure 3 Conversion of [^{13}C]18:3n-3 to longer chain PUFA in Con. A simulated PBMCs from men (n=12) and women (n=14) over 72 hours. *Indicated means that were significantly different (P < 0.05) between sexes by Student's unpaired t test.

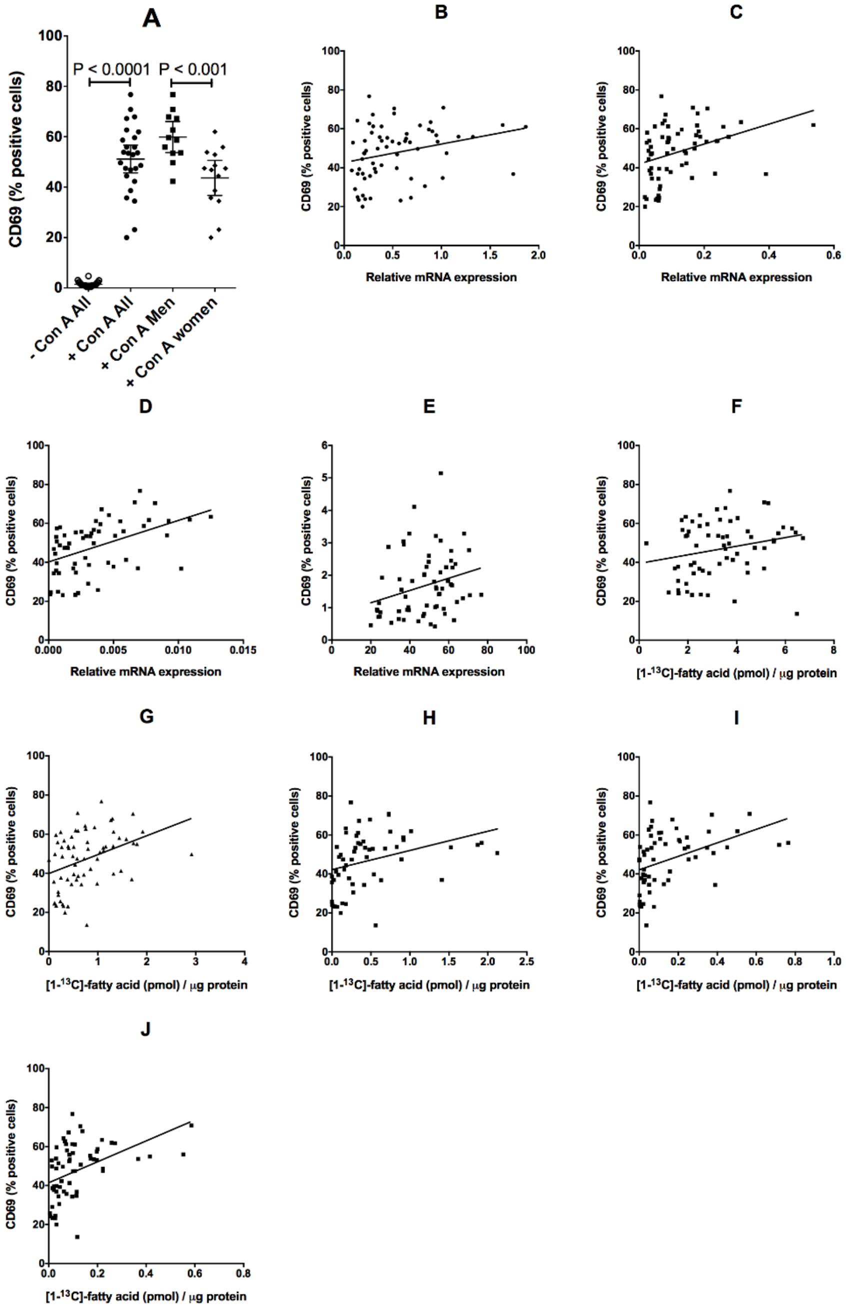

Supplementary Figure 4. Associations between FADS and ELOVL mRNA expression or [13C] enrichment of n-3 PUFA, and CD69 expression in Con. A –stimulated PBMCs. (A) CD69 expression in control and Con. A –stimulated cells in all subjects (n = 26) and in men (n=12) and women (n = 14) separately. Bars are mean and 95% confidence interval. Statistical testing for the effect of cell activation was by Students paired t test, while CD69 expression in cells from men and women were compared by unpaired t test. B – E Linear regression analysis of the relationship between CD69 expression and (B) FADS2, (C) FADS1, (D) ELOVL4 and (E) ELOVL5 mRNA expression in Con. A –stimulated cells. F - J Linear regression analysis of the relationship between CD69 expression and [13C] enrichment of (F) 18:3n-3, (G) 20:3n-3, (H) 20:4n-3, (I) 20:5n-3 and (J) 22:5n-3 in Con. A –stimulated cells.

Raw data generated for the study

Raw data are presented below in the order in which they appear in figures in the main document.

Figure 2 Synthesis of PUFA by PBMCs and Jurkat cells

Figure 2A

	Mass of labelled fatty acid (pmol/µg protein)																									
Subjects	1	2	3	4	5	6	7	8	9	10	11	12	13	14	15	16	17	18	19	20	21	22	23	24	25	26
18:3n-3	7.843	1.063	3.060	0.926	1.699	2.012	1.424	2.007	1.300	1.128	0.936	1.028	3.749	1.049	1.022	1.860	2.140	0.800	1.892	1.133	1.011	0.322	2.078	1.088	0.550	1.096
	Unstimulated PBMCs																									
18:4n-3																										
20:3n-3	0.089	0.075	0.000	0.000	0.000	0.000	0.002	0.106	0.058	0.036	0.020	0.022	0.251	0.068	0.123	0.099	0.139	0.000	0.116	0.000	0.064	0.044	0.180	0.143	0.053	0.055
20:4n-3	0.000	0.106	0.000	0.000	0.000	0.000	0.000	0.000	0.000	0.021	0.000	0.000	0.024	0.106	0.051	0.000	0.000	0.000	0.000	0.000	0.079	0.011	0.000	0.000	0.000	0.046
20:5n-3	0.010	0.045	0.000	0.000	0.010	0.003	0.001	0.025	0.056	0.003	0.000	0.008	0.015	0.005	0.006	0.029	0.050	0.000	0.008	0.009	0.025	0.008	0.000	0.002	0.004	0.012
22:5n-3	0.014	0.015	0.010	0.002	0.010	0.011	0.005	0.020	0.046	0.004	0.017	0.001	0.007	0.012	0.013	0.031	0.019	0.008	0.027	0.009	0.000	0.016	0.016	0.005	0.019	0.026
22:6n-3																										
	Stimulated PBMCs																									
Subject	1	2	3	4	5	6	7	8	9	10	11	12	13	14	15	16	17	18	19	20	21	22	23	24	25	26
18:3n-3	6.476	4.744	4.019	2.345	2.609	4.039	4.743	3.726	1.210	1.973	2.027	2.122	3.913	3.427	4.453	5.133	3.688	3.591	2.799	3.242	1.914	2.820	2.870	3.319	2.482	3.537
18:4n-3																										
20:3n-3	0.774	0.721	0.535	0.274	0.103	0.755	0.576	1.072	0.112	0.395	0.178	0.598	0.329	0.406	0.788	0.588	2.913	0.850	0.144	1.161	0.507	0.390	0.462	0.546	0.466	1.299
20:4n-3	0.561	0.143	0.179	0.446	0.000	0.000	0.892	0.242	0.181	0.062	0.336	0.000	0.112	0.524	1.920	0.729	0.000	0.158	0.314	1.525	0.425	0.069	0.000	1.015	0.916	0.488
20:5n-3	0.117	0.108	0.008	0.222	0.073	0.067	0.222	0.097	0.027	0.030	0.085	0.016	0.032	0.012	0.553	0.586	0.058	0.052	0.032	0.367	0.186	0.027	0.040	0.258	0.200	0.138
22:6n-3																										
Replicates	Jurkat cells																									
18:3n-3	0.841	0.743	0.932	0.957	1.015	0.954	0.846	1.044	0.958																	
18:4n-3	0.000	0.351	0.466	0.490	0.497	0.000	0.427																			
20:3n-3	0.396	0.303	0.446	0.461	0.439	0.427	0.421	0.391	0.361																	
20:4n-3	3.254	2.210	3.800	4.071	3.351	3.277	2.906	3.129	2.623																	
20:5n-3	7.303	5.257	8.162	8.682	7.515	7.520	6.644	6.084	6.436																	
22:5n-3	12.803	9.032	14.320	15.063	12.268	12.362	10.702	10.738	11.622																	
22:6n-3	0.040	0.055	0.037	0.084	0.021	0.072	0.016	0.064	0.074																	

Figure 2B

	Proportion of n-3 PUFA (%)				
Subjects	1	2	3	4	5
	Untreated PBMCs				
18:3n-3	49.2	47.8	48.8	48.5	43.3
18:4n-3					
20:3n-3	7.7	7.5	8.1	8.9	8.4
20:4n-3	2.3	1.4	1.5	1.5	1.5
20:5n-3	5.5	2.6	2.6	2.4	3.3
22:5n-3	20.1	23.3	22.2	22.8	25.3
22:6n-3	15.3	17.3	16.8	17.3	17.1
	PBMCs treated with SC26196				
Subjects	1	2	3	4	5
18:3n-3	49.04	51.78	51.33	47.14	42.74
18:4n-3					
20:3n-3	8.87	10.38	9.56	9.9	8.88
20:4n-3	0.94	0.58	0.61	0.67	0.54
20:5n-3	3.73	2.35	2.8	3.53	3.18
22:6n-3	15.77	15.01	15.23	17.12	18.8

Figure 2C

	Mass of labelled fatty acid (pmol/µg protein)				
Subjects	1	2	3	4	5
	Untreated PBMCs				
18:3n-3	5.653	7.139	6.353	2.985	4.230
18:4n-3	3.551	4.086	5.558	3.396	4.538
20:3n-3	1.337	1.502	2.309	0.976	1.617
20:4n-3	4.205	4.384	6.056	3.116	4.023
20:5n-3	7.264	7.217	8.725	7.048	7.688
22:5n-3	16.385	15.598	19.361	15.354	17.054
22:6n-3	0.119	0.099	0.122	0.106	0.115
	PBMCs treated with SC26196				
Subjects					
18:3n-3	6.596	7.356	10.003	7.665	6.495
18:4n-3	4.059	4.273	4.280	3.574	
20:3n-3	3.334	2.365	3.458		2.364
20:4n-3	2.805	2.716	3.164	2.655	2.460
20:5n-3	5.851	5.521	6.898	5.596	6.200
22:6n-3	12.509	11.470	13.708	11.853	12.195

Figure 3 mRNA expression of gene involved in PUFA synthesis

	Relative mRNA expression																											
Subjects/CR	1	2	3	4	5	6	7	8	9	10	11	12	13	14	15	16	17	18	19			20	21	22	23	24	25	26
	Figure 3A																											
U PBMCs	0.07	0.46	0.51	0.05	0.08	0.3	0.78	0.07	0.15	0.2	0.02	0.37	0.13	0.08	0.04	0.41	0.06	0.19	0.17	0.08	0.35	0.7	0.11	0.74	0.04	0.42	0.1	0.58
S PBMCs	0.21	1.32	1.63	0.08	0.27	0.58	1.81	0.09	0.21	0.19	0.19	1.05	0.21	0.55	0.52	0.63	0.26	0.26	0.51	0.3	0.87	1.18	0.76	1.02	0.3	0.5	0.37	0.91
Jurkat	20.22	14.3	15.28	19.12	17.2	15.71	9.96	27.14																				
	Figure 3B																											
U PBMCs	0.03	0.12	0.08	0.02	0.06	0.1	0.04	0.06	0.11	0.01	0.08	0.03	0.03	0.05	0.07	0.03	0.11	0.08	0.04	0.03	0.06	0.02	0.12	0.03	0.05	0.06	0.06	0.1
S PBMCs	0.04	0.28	0.54	0.03	0.06	0.14	0.02	0.03	0.02	0.03	0.14	0.09	0.04	0.21	0.24	0.07	0.07	0.18	0.08	0.13	0.19	0.1	0.17	0.15	0.09	0.07	0.18	0.37
Jurkat	3.35	1.5	2.55	1.97	3.63	5.69	2.73	2.71	4.59	3.71																		
	Figure 3C																											
U PBMCs	0.003		0.001	0.001	0.003	0.001	0.000	0.000	0.001	0.001	0.001	0.001	0.001	0.001	0.001	0.003	0.001	0.005	0.002	0.001	0.002	0.001	0.001	0.001	0.000	0.001	0.001	0.001
S PBMCs	0.008	0.005	0.007		0.021	0.004	0.004	0.006	0.002	0.007	0.003	0.003	0.003	0.007	0.001	0.004	0.011	0.003	0.001	0.001			0.001		0.001	0.001	0.001	0.000
Jurkat	0.307	0.379	0.633	0.295	0.350	0.440	0.369	0.833	0.251	0.220																		
	Figure 3D																											
U PBMCs	0.92	1.22	1.19	0.72	1.47	0.89	1.58	0.79	1.08	1.37	0.93	1.15	1.21	0.76	1.24	1.24	0.85	1.02	1.19	1.45	0.62	0.78	0.75	0.92	1.31	1.26	1.23	1.14
S PBMCs	0.89	3.07	2.24	0.94	1.55	0.94	2.26	0.42	1.05	0.46	0.58	2.03	2.01	0.74	2.77	1.59	1.4	0.61	3.29	1.29	1.83	5.14	2.41	1.39	4.11	1.63	2.09	2.25
Jurkat	11.83	9.56	15.88	12.82	20.53	37.7	14.42	12.46	15.56	11.21																		
	Figure 3E																											
Jurkat	0.008	0.009	0.011	0.010	0.007	0.012	0.010	0.009	0.007	0.004																		

UPBMC, unstimulated PBMCs; SPBMC, stimulated PBMCs; CR, culture replicates

Figure 4 The effect of SC26196 on the proliferation of PBMCs and Jurkat cells

	Cell proliferation parameters																											
Subjects/CR	1	2	3	4	5	6	7	8	9	10	11	12	13	14	15	16	17	18	19			20	21	22	23	24	25	26
	Figure 4A: Proliferation index of PBMCs																											
Control	2.69	2.62	2.68	2.68	2.62	2.61																						
Treated	2.53	2.48	2.38	2.44	2.39	2.41																						
	Figure 4B: Division index Of PBMCs																											
Control	1.79	1.7	1.78	1.75	1.73	1.75																						
Treated	1.6	1.54	1.48	1.53	1.58	1.63																						
	Figure 4C: Proportion of PBMCs that underwent cell division																											
Control	72.2	71.6	74.3	73.3	72.2	72																						
Treated	68.2	67.5	68.3	68.4	69.3	71.2																						
	Figure 3D: Proliferation of Jurkat cells																											
Control	5.94	5.89	5.77	5.90	5.79	6.01	5.85	5.64	5.41	5.94																		
Treated	5.85	5.94	5.51	5.74	5.24	5.58	5.51	5.50	5.62	5.85																		

Figure 6 *FADS2* DNA methylation in PBMCs

Figure 6A 5' Regulatory region; Unstimulated PBMCs

	Methylation (%)																																
Subject	1	2	3	4	5	6	7	8	9	10	11	12	13	14	15	16	17	18	19	20	21	22	23	24	25	26	27	28	29	30	31	32	33
CpG																																	
-1661	94.4	94.5	95.2	94.6	94.6	94.6	95.0	95.5	96.5	96.2	95.2	95.0	96.7	95.6	95.8	96.5	94.7	94.7	95.1	96.1	95.8	94.6	93.9	95.1	95.3	94.2	93.5	94.6	95.8	94.6	96.0	95.8	96.4
-1655	83.6	87.1	85.1	86.2	87.2	89.8	89.4	88.0	89.6	88.3	88.0	87.5	87.4	86.8	86.5	87.0	87.6	88.1	88.9	89.7	87.8	83.4	85.1	86.6	88.2	82.9	77.9	87.0	88.2	85.5	87.1	86.7	87.8
-1337	77.1	76.1	77.9	73.0	74.6	80.7	81.4	76.2	78.8	82.4	83.0	82.0	84.2	78.3	81.7	78.7	78.7	80.7	80.8	80.0	81.8	74.6	75.8	82.1	81.9	77.3	74.0	76.1	80.1	73.8	75.4	82.0	79.3
-1278	62.1	68.6	59.6	63.4	66.5	73.0	72.4	60.9	64.8	65.6	64.1	70.3	68.0	65.5	67.9	72.4	71.6	69.1	71.5	63.4	68.6	51.2	64.4	69.1	59.8	52.3	55.9	70.7	72.1	68.9	71.9	64.1	65.0
-1156	58.8	58.7	57.6	50.0	50.0	62.9	59.1	49.7	52.6	55.4	56.6	65.1	71.0	61.8	51.7	68.9	58.8	53.8	57.4	53.1	62.9	39.0	49.9	58.9	47.0	50.3	43.1	55.5	59.1	53.8	55.9	54.6	53.7
-1119	24.0	24.2	20.7	17.1	21.3	27.6	30.3	17.2	17.7	13.8	16.5	23.5	28.1	19.5	15.0	19.4	24.6	14.6	22.2	20.2	22.5	10.2	23.4	23.0	13.7	19.8	16.8	16.1	20.6	18.1	15.5	22.1	
-1112	37.2	33.6	32.9	27.8	29.6	37.8	38.4	27.3	27.6	24.8	25.0	38.2	41.3	33.8	27.6	32.0	37.7	25.3	32.5	29.1	38.0	15.9	32.4	24.9	23.0	28.0	28.2	28.4	31.0	29.1	34.6	30.8	
-1101	31.5	34.0	27.5	25.0	29.6	36.6	36.1	25.5	25.1	20.4	21.7	37.2	37.7	32.0	28.2	30.3	34.5	24.2	27.1	26.6	31.6	17.0	34.0	29.4	19.4	25.5	25.4	24.6	29.3	25.6	27.8	26.4	
-1071	45.8	47.8	39.0	40.0	41.2	52.5	49.8	37.5	43.4	38.4	43.6	54.4	56.1	50.9		48.8	52.1	41.8	50.5	46.9	53.7	26.5	44.6	51.7	42.8	41.6	37.9		47.4		43.8	46.8	
-1067	39.1	37.5	36.6	28.5	33.4	41.2	45.0	31.7	33.5	28.5	26.0	44.3	41.2	35.8		41.0	39.4	33.6	37.1	29.7	5.5	17.2	36.0	41.6	27.4	30.1	29.8		35.1		37.7	34.5	
-1056	50.0	48.1	46.0	42.3	42.8	52.2	52.8	39.4	51.0	40.6	37.7	52.8	57.8	47.5		45.5	55.5	44.1	48.6	51.5	60.7	27.3	48.3	52.6	39.6	1.5	37.4		47.2		49.0	47.1	
-1013	57.5	62.9	61.5	57.9	30.3	72.2	67.0	57.2	66.8	55.2	65.1		60.9	63.1	55.6	63.1	65.9	62.6	69.0	63.8	73.7	48.8	60.8	65.2	56.6	52.6	53.0	61.6	72.3	67.6	61.6	60.7	62.2
-980	41.6	43.2	39.0	36.6	20.9	48.7	47.5	34.8	36.6	28.8	32.9		42.5	40.5	32.1	36.9	39.9	35.0	40.8	35.0	43.0	21.0	43.4	41.8	27.4	31.2	32.2	39.1	40.7	36.5	40.7	37.1	39.0
-975	47.5	54.4	46.0	45.9	24.1	62.7	56.2	41.3	51.2	39.1	48.2		50.6	56.8	41.2	50.7	54.3	46.7	55.4	48.8	58.9	31.9	48.3	57.3	41.1	39.9	43.7	51.5	60.7	44.8	52.5	53.4	51.0
-914	19.5	18.6	14.6		15.8	21.6	25.2	13.8	12.9	14.3	12.9	20.8	20.9	16.0	14.9	16.2	15.5	13.9			16.1	5.5	17.1	16.4	11.0	0.7	10.3	12.0	15.6	14.3		14.9	
-871	26.2	24.0	19.0	16.7	24.0	23.3	26.8	13.7	14.5	14.3	12.1	29.1	32.1	20.8	19.7	19.2	23.4	19.8	22.7	13.8	22.4		28.1	23.8	10.7	14.3	14.8	18.0	17.3	20.3	20.1	17.2	20.6
-869	26.5	24.8	16.0	15.2	21.1	27.1	27.5	13.0	16.2	12.6	12.3	29.6	32.2	18.6	17.4	21.2	25.1	19.9	31.2	14.5	22.1		28.8	24.0	0.8	11.6	17.1	18.6	22.3	1.3	21.4	21.8	18.0
-855	28.0	25.9	21.3	18.4	23.1	25.6	29.6	15.3	18.5	18.0	14.7	33.6	32.1	24.6	17.0	21.5	24.2	18.7		17.1	23.3		11.0	24.9	15.1		18.8	18.3	22.2	21.6	22.0	22.9	19.3
-817	22.4	23.2	19.5	15.4	19.0	22.2	21.2	12.4	12.1	11.5	10.7	23.6	27.4	15.1	14.6	18.9	22.1	13.5	18.1	14.3	17.6		23.5	34.5	23.2	27.2	34.7	16.6	17.2	17.1	16.6	14.6	17.6
-806	28.8	28.6	19.2	18.9	21.4	29.8	27.3	14.3	16.8	13.4	12.5	25.3	30.8	25.8	18.7	24.7	28.7	19.4	24.0	16.7	22.6		25.5	20.3	13.9	16.9	15.8	19.0	21.8	20.7	21.6	22.4	21.1
-775	29.3	30.1	21.7	24.2		40.1		17.2	19.2		13.8	38.8	34.3	27.2	24.1	30.3			29.3		29.3		30.2	31.8	29.8	19.5	17.6	24.1	24.3	26.6	25.8	28.5	
-718	4.5	9.3	9.7	6.5	9.7	9.7	9.8	8.1	6.6	8.5	4.8	10.4	13.0	8.1	8.0	6.3	10.0	7.3	9.9	4.4	13.6		11.2	10.7	5.6	5.3	7.5	6.3		9.8	6.2	7.6	
-686	11.5	9.9	7.2	7.5	10.4	9.5	12.4	5.8	6.1	5.1	4.3	12.7	11.9	7.3	7.3	9.9	9.0	7.7	8.0	6.3	9.3		12.0	10.6	5.3	6.4	6.5	7.2	6.8	8.5	8.4	7.1	7.6
-669	18.9	16.1	17.1	12.9	14.6	14.4	18.5	11.0	14.4	15.8	13.9			17.3	12.8	13.9	15.1	11.0	19.1	13.1	17.0		19.3	16.7	13.7	13.0	13.0	15.1	11.5	10.9	13.8		12.4
-667		15.1		12.8	11.7	15.3	14.0	10.5	6.2	9.1	5.6		15.1	10.8	9.5	12.6	13.7	11.2	11.4	7.6	19.0						11.1	10.1	10.9	11.5	14.0	14.1	9.0
-374	1.5	0.0	1.3	0.7	1.8	1.6	1.7	2.4	1.4	2.1	0.0		0.0	1.5	1.9	0.0	0.0	0.0	1.0	2.1	1.5	0.0	1.1	0.0	1.8	0.0	0.8	0.7	0.0	0.0	0.0	1.6	2.0
-368	1.7	1.1	0.7	1.0	1.7	1.6	0.0	1.9	2.7	0.9	1.2	0.0	0.0	2.8	1.6	1.7	1.6	0.0	0.9	2.2	0.8	0.0	0.9	0.9	1.0	2.3	1.0	2.8	0.0	0.0	1.8	1.3	0.8
-356	7.3	7.6	7.3	3.6	7.8	8.8	4.4	11.8	12.2	7.5	10.1	0.0	4.7	8.3	9.2	11.4	5.5	6.9	5.9	7.5	8.4	6.0	11.3	6.6	6.9	4.8	6.4	7.7	7.7	5.9	5.3	7.6	7.5
-344	3.8	2.4	3.6	3.1	3.8	5.5	4.2	8.1	6.5	3.4	4.1	0.0	1.9	3.4	5.0	7.0	5.1	3.3	2.9	6.7	5.9	6.1	3.6	4.0	3.8	3.1	4.5	3.4	3.9	3.2	3.3	4.7	4.4
-336	3.0	2.3	2.9	1.9	2.3	2.3	1.0	4.7	4.9	2.4	4.3	0.0	1.6	3.8	3.3	4.0	3.2	2.6	1.0	4.0	2.8	2.2	2.9	2.3	2.5	1.3	2.0	1.6	3.0	2.7	1.5	4.2	1.9
-334	3.5	0.0	2.0	4.0	2.1	4.0	2.6	5.4	1.6	2.9	3.0		1.3	2.1	3.5	2.2	0.0	0.0	2.9	3.1	4.8	0.0	2.6	0.0	1.7	2.7	1.1	0.0	2.2	2.4	2.6	3.0	0.0
-258	0.8	0.0	1.8	0.9	0.7	0.9	0.7	1.1	0.8	1.4	0.9	1.1	0.6	1.0		1.0	1.2	0.0	0.8	0.0	1.0	0.0	1.7	0.8	1.2	0.7		3.0				1.1	1.9
-253	1.6	2.6	2.7	1.7	2.3	2.6	1.2	1.7	1.0	2.2	0.9	1.5	3.1	1.9		1.6	1.2	0.0	1.9	1.1	1.7	0.0	1.4	1.3	1.3	0.8	1.6	2.5	0.0			2.1	2.1
-250	0.7	0.9	2.5	1.0	1.0	1.0	0.9	1.1	0.8	1.2	0.7	0.8	1.1	1.2	0.0	1.1	1.2	0.0	0.8	0.0	0.6	0.0	1.1	0.9	1.0	0.9	2.4	3.6	1.3		0.0	1.1	4.4
-248	0.9	1.2	2.0	1.0	0.8	1.0	0.7	0.6	0.5	1.3	0.6	1.3	1.1	0.9	1.5	1.3	0.8	0.0	0.7	0.0	0.5	0.0	1.0	0.6	0.7	0.8	2.2	3.5	0.7		0.0	0.7	
-244	1.8	1.8	4.1	2.1	1.9	2.6	1.5	1.7	1.8	2.0	1.2	1.8	2.1	2.4		1.4	1.6	0.0	1.8	0.0	1.6	0.0	2.5	1.8	1.1	2.0	3.4	5.6	1.5		0.0	1.9	
-230	1.2	0.8	0.9	0.9	0.8	1.6	0.9	2.3	2.7	1.7	1.3	0.8	0.5	1.3	1.5	1.7	1.6	0.7	0.9	2.4	1.4	0.0	1.2	1.4	1.0	1.6	0.9	1.1	1.1	2.0	0.8	1.9	1.0
-218	5.5	5.1	7.1	6.4	4.4	6.4	5.3	8.2	8.8	6.0	6.8	4.3	4.6	5.4	6.6	6.6	4.0	6.1	5.0	9.2	8.0	4.5	7.0	5.7	5.3	6.0	5.1	5.6	5.2	5.9	5.1	5.6	7.2
-205	7.6	7.4	7.2	6.3	3.8	9.5	6.6	12.0	13.2	6.8	10.0	3.2	3.6		8.4	8.4	6.4	6.8	7.3	12.6	10.2	6.5	8.5	6.8	6.9	7.1	5.3	6.4	7.0	7.3	6.4	9.5	7.3
-201	1.9	1.6	1.0	1.1	0.5	1.9	1.6	3.1	2.2	1.6	1.8	0.9	0.7		1.6	2.1	1.2	1.3	1.9	3.4	2.8	1.4	1.8	1.5	1.8	1.5	1.1	1.7	1.4	2.2	1.5	2.0	1.4
-198	1.0	0.0	1.2	0.7	0.7	1.4	1.1	1.0	1.3	1.0	0.0	0.0	0.7		1.0	0.0	1.1	0.0	0.6	0.0	1.0	0.0	0.7	0.7	1.0	0.7	0.4	0.8	0.0	0.9	0.0	1.5	1.0
-191	5.1	4.9	3.5	5.3	1.4	7.0	4.2	8.0	8.4	4.7	6.5	0.0	1.4		6.3	5.6	3.5		3.6	7.0	7.1	3.8	5.5	4.3		5.5	2.8	4.7		4.4	4.5	6.8	5.5
-189	1.6	0.0	1.8	1.4	1.0	2.2	1.5	2.9	2.6	1.6	1.6	0.0	0.0		1.5	1.2	1.8		1.1	1.3	0.0	0.0	1.9	1.5	1.0	1.5	0.8	1.0		1.8	1.0	1.5	0.9
-172	4.6	4.6	4.7	4.0	1.4	6.5	3.4	9.1	9.5	3.8	5.8	1.4	1.1	4.2	5.3	5.8	3.6	2.8	3.0	7.5	6.5	2.6	5.2	4.0	4.4	5.0	4.1	3.2	3.8	3.6	2.8	8.2	4.5
-167	3.6	1.0	2.6	2.6	0.7	4.7	2.3	5.6	5.5	2.5	4.3	0.0	1.5	2.3	3.6	1.8	2.2	0.8	2.4	3.8	0.0	0.9	2.7	2.4	2.5	0.0	2.1	1.7	1.7	2.8	1.3	3.0	1.5
-157	5.5	4.4	4.8	5.1	2.1	8.8	3.9	10.7	10.3	3.9	6.7	1.5	1.6	4.4	6.3	5.4	4.0	3.9	3.5	8.9	7.7	3.2	4.9	5.1	4.5	4.4	3.8	3.8	4.4	4.1	4.1	7.0	5.1
-149	1.6	0.0	1.2	1.0	0.7	2.5	1.0	3.5	3.0	1.3	0.9	0.0	0.7	0.9	2.2	0.9	1.2	0.0	1.4	1.5	1.5	0.0	2.1	1.1	1.4	1.5	1.4	1.6	0.9	1.8	0.6	2.2	1.3
-133	2.7	2.1	2.5	2.4	1.8	3.0	2.4	3.3	3.5	2.6	3.0	2.1	1.5	2.1	3.2	3.0	3.4	2.0	2.6	3.0	3.5	2.0	3.5	2.1	2.3	2.8	2.6	2.2	2.4	2.6	2.2	2.5	1.9
-117	1.2	0.0	2.4	0.9	1.5	1.2	1.1	1.9	1.4	1.4	0.8	0.9	1.2	0.8	1.3	0.0	1.7	0.0	0.9	0.0	1.3	0.0	1.3	0.9	1.4	0.6	1.4	1.2	0.9	1.3	0.5	1.2	1.3
-64	1.8	1.4		1.5	1.9	1.7	1.7	1.9	1.6	2.0	1.4	2.2	1.7	2.1	7.2		1.9	1.4	1.9	1.2	2.2	1.2	1.9	1.5	1.8	1.1	4.2	2.4	1.8	2.2		2.1	2.7
-50	2.8	2.5		2.4	2.8	2.9	2.8	2.3	2.6	2.2	1.6	2.8	3.2	2.8	4.1		2.4	2.7	2.5	2.9	2.7	2.5	3.1	2.5	2.4	8.6	4.6	3.6	6.0	4.3		2.5	6.6
-18	3.3	3.6	4.9	3.3	3.7	3.8	3.8	3.6	2.8	3.3	2.1	3.0	3.8	4.0	3.2	3.1	4.0	3.8	3.2	3.5	3.3	2.8	3.6	3.7	3.4	2.9	4.6	6.1	3.3	3.1	3.4	3.3	3.5

Figure 6A 5’ Regulatory region; Stimulated PBMCs

	Methylation (%)																																
Subject	1	2	3	4	5	6	7	8	9	10	11	12	13	14	15	16	17	18	19	20	21	22	23	24	25	26	27	28	29	30	31	32	33
CpG																																	
-1661	95.7	94.3	93.5	93.6	94.3	94.1	94.1	94.6	94.6	95.6	95.7	93.1	94.2	95.7		94.7	94.6	96.3	95.0	94.6	95.6	94.2	93.9	95.0	95.0	94.5	94.6	94.6	96.9	94.8	96.9	94.5	94.8
-1655	89.4	87.4	88.1	86.2	85.7	86.6	86.4	81.5	86.6	88.0	84.3	82.6	86.9	90.5		88.9	88.2	87.4	87.1	84.7	87.9	84.2	86.8	84.1	89.6	87.4	88.8	89.3	91.9	90.1	89.0	88.7	85.5
-1337	75.6	74.1	71.8	69.5	71.5	70.8	73.7	67.4		79.8	74.3	76.5	75.1	84.1	73.1	76.5	76.0	76.3	68.5	73.7	79.2	77.2	78.1	77.0	73.6	81.0	76.5	76.9	77.4	76.6	81.3	77.6	83.9
-1278	77.8	73.8	68.5	67.9	67.9	72.4	70.1	59.4		66.4	60.3	68.5	82.2	77.9	71.2	77.6	77.9	73.2	72.0	65.5	71.6	56.2	69.7	71.1	68.7	68.4	72.3	73.7	75.7	67.9	73.5	70.4	70.2
-1156	68.4	65.7	66.7	60.1	62.5	73.1	71.2	67.1		64.1	57.7	73.0	78.4	64.6	61.7	71.5	67.4	71.3	28.6	60.3	65.1	51.8	69.0	71.8	59.6	65.7	71.8	62.8	68.5	56.0	71.8	69.0	59.3
-1119	26.7	27.7	30.9	22.3	29.8	30.5	30.0	19.4	15.6	14.7	14.6	25.0	30.4	18.6	20.5	27.1	21.7	26.7	23.9	16.8	21.3	11.6	27.4	23.0	16.7	20.0		23.6	22.3	17.7	24.1	24.1	21.9
-1112	46.2	43.4	38.5	32.7	35.4	42.6	43.5	31.3	24.8	25.2	24.8	44.0	51.9	36.4	30.1	40.5	35.4	34.2	38.2	30.3	41.7	19.4	41.7	41.0	27.8	33.8		35.4	37.3	27.6	36.6	38.9	32.8
-1101	40.5	40.6	37.9	33.0	34.7	39.3	45.4	27.4	25.4	23.1	18.6	38.9	43.8	32.0	23.8	32.4	31.9	31.9	30.7	23.1	36.9	19.0	42.0	27.2	24.0	30.7		30.3	27.8	28.7	29.8	34.8	26.2
-1071	52.9	53.8	44.6	44.6	47.1	55.7	57.0	42.0	41.9	39.7	45.4	47.0	55.6			53.5	52.2	49.8	53.8	46.8	56.6	29.7	50.5	53.4	42.3	45.4		52.8	53.8	42.7	51.4	55.6	50.0
-1067	46.2	46.5	37.1	32.9	40.6	45.3	49.5	37.1	32.4	34.1	29.2	45.4	49.9			37.2	43.7	41.9	40.8	31.9	42.5	20.8	44.7	44.5	28.6	36.5		40.0	40.3	35.4	37.8	41.5	36.1
-1056	51.9	54.2	50.0	50.0	48.7	56.3	56.9	42.8	38.0	43.7	46.2	58.7	56.9			46.8	54.7	46.0	50.4	46.3	55.5	31.6	55.3	55.6	41.2	45.1			51.7	43.3	51.4	56.7	51.2
-1013	70.2	73.7	69.1	61.8	61.9	72.5	72.5	65.2	65.9	65.4	47.2	75.3	78.3	75.1	63.5	73.1	70.8	70.0	72.4	62.1	72.9	62.1	70.2	73.8	68.5	74.1	74.7	67.8	77.8	56.1	63.8	71.9	59.8
-980	55.0	49.7	45.6	41.8	46.5	59.7	56.8	40.2	34.9	35.6	20.4	48.8	58.0	49.4	37.1	44.7	44.5	46.7	48.6	38.2	48.4	27.8	55.5	47.7	35.9	42.9	46.9	44.0	45.7	37.6	40.1	48.5	38.8
-975	64.0	66.4	54.9	51.0	56.4	68.2	65.0	50.9	51.1	47.4	34.8	65.9	72.4	65.7	49.4	64.2	62.3	61.2	63.3	53.7	68.4	39.5	63.2	66.5	55.0	59.4	64.8	60.3	66.7	49.5	54.0	66.9	52.9
-914	24.3	20.8	19.4	17.1	25.6	19.9	19.0	14.1	12.5	12.8	14.0	27.0	24.4	18.2	13.8	21.7	26.0				15.0	8.4	21.1	16.9	11.6	15.5	12.0	25.4	16.5	19.6	16.3	21.4	16.2
-871	27.4	26.4	19.6	17.7	27.6	30.2	33.4	16.2		13.3	22.0	24.5	34.6	22.1	20.0	23.5	22.7	26.1	29.7	17.0	24.4		34.9	26.3	16.2	16.3	20.5	21.2	22.3	22.7	23.7	26.6	23.0
-869	25.1	27.0	15.9	21.0	24.6	29.3	30.4	19.6		19.8	18.8	26.3	35.2	24.1	20.1	23.5	27.5	27.3	29.3	17.1	27.1		32.5	25.6	14.2	17.7	19.9	19.1	25.7	25.3	22.8	26.7	23.5
-855	28.7	26.9	17.3	23.3	28.0	31.1	36.4	15.0		17.4	20.9	27.6	34.5	25.8	23.7	19.2	26.6	26.6	30.9	16.0	28.5		33.9	30.6	15.0	18.8	21.0	20.4	24.9	28.0	26.9	24.3	21.7
-817	18.8	25.3	15.6	16.1	23.1	26.5	21.2	15.0	15.3	15.8	12.9	24.1	28.8	17.3	14.3	17.8	21.2	20.0	17.3	15.7	19.2	9.4	28.8	11.0	13.7	15.0	20.6	17.0	18.1	17.7	17.6	20.4	19.5
-806	22.4	28.4	19.7	18.4	30.5	31.8	26.0	16.6	17.6	17.9	13.9	28.2	37.5	23.9	18.5	25.3	27.9	26.8	21.8	14.9	25.2	8.1	34.3	21.2	15.4	16.6	23.5	22.3	25.3	23.6	20.5	27.1	23.3
-775	34.6	32.5	25.4		34.0	40.1	34.1	24.0	18.3	18.3	16.0	40.5	43.6	32.6	22.3	26.9	30.2	30.9	29.5	25.4	30.4	7.1	40.2	30.1	19.9	22.7	27.6	32.0				30.9	29.2
-718	13.6	10.8	9.0	7.1	11.1	11.6	14.2	6.3	7.9		7.9	16.5	14.6	8.2	9.7	9.6	8.8	8.2	9.3	8.4		5.4	12.7	12.0	6.1	7.3	8.9	6.2	6.4		9.1	9.1	8.1
-686	12.5	9.9	7.9	8.7	12.1	14.2	14.3	6.5	6.9	5.9	6.0	14.5	12.1	7.1	8.6	11.3	9.9	9.7	9.2	6.1	8.8	2.9	16.4	10.9	5.9	7.4	7.9	8.4	7.0	8.3	10.2	8.6	9.8
-669	19.3	16.4	16.5	13.9	22.0	15.4	17.1	18.1	15.6	14.2	9.9	20.1	15.8	13.1	14.0	13.1	18.4	15.0	17.5	8.9	15.3	10.3	19.9	18.1	11.1	12.6	16.9	15.5	7.6	15.6	15.9	19.1	18.5
-667	17.3	16.2	11.1	13.4	16.0	16.4	17.2	14.2	8.2	11.3	11.1	20.2	22.2	12.4	14.5	11.2		15.2	17.1	10.0	18.2		24.1	16.4	11.4	14.1	11.7	16.5	10.6	13.9	15.0	15.3	
-374	0.9	1.5	0.0	0.0	1.4	0.0	1.3	0.5	0.0	0.8	0.0	2.2	0.7	0.0	2.2	0.0	4.1	1.3	1.2	0.0	0.9	4.3	1.6	0.9	0.0	2.1	1.4	1.6	3.6	5.1	0.0	9.1	0.0
-368	1.8	2.3	0.0	0.0	0.0	0.0	0.0	0.7	1.1	1.1	0.0	1.3	1.0	2.6	0.8	2.2	0.0	1.8	1.8	0.0	0.7	1.8	1.8	0.0	1.5	2.0	0.0	1.0	2.4	2.6	0.0	0.0	0.0
-356	5.3	6.0	5.7	6.0	7.0	6.5	4.6	6.3	6.7	5.8		6.8	8.1	7.4	10.3	5.8	16.0	5.9	5.9	10.7	8.7	8.0	6.1	4.1	4.9	10.5	6.5	7.8	17.0	13.1	9.1	9.2	14.2
-344	2.7	4.3	4.3	3.3	3.0	3.0	4.8	2.8	5.8	2.2	0.0	3.5	2.2	3.4	5.9	3.5	7.5	2.7	6.1	6.1	4.1	6.8	4.2	3.8	2.0	4.3	3.2	2.9	5.1	5.5	0.0	2.9	5.9
-336	2.2	1.4	5.4	1.5	3.5	2.4	2.0	1.2	4.5	1.5	0.0	1.9	1.0	3.9	2.9	3.3	4.3	3.5	1.4	1.6	2.3	3.7	5.3	2.7	0.0	1.4	2.1	2.7	4.1	3.9	0.0	4.8	5.4
-334	1.3	2.9	1.2	2.7	2.4	2.5	2.1	3.6	2.8	3.4	0.0	3.3	2.3	2.4	2.1	0.0	3.7	2.1	0.0	2.9	1.9	2.3	2.8	2.7	0.0	2.0	5.1	1.8	1.7	2.4	0.0	1.8	0.0
-258	1.7	2.0	1.7	1.1	2.5	3.4	0.9		3.2	2.5	3.1		4.7	2.4	2.8	1.0	1.0			0.9	1.2		2.0		0.8	1.4	2.9	0.9	0.8	1.2	0.0	1.0	0.0
-253	2.4	2.6	1.4	3.1		1.5	0.6	3.1	6.6	5.2		2.0	2.7	1.9	2.9	3.3	1.8	1.3	0.0	2.0	1.1		3.0		1.8	1.5	0.0	1.9	0.9	1.7	2.0	2.4	1.8
-250	1.9	2.9	1.7	1.1	2.6	3.4	1.4	0.7	2.8	3.9	2.2			2.3	0.0	0.6	1.3	2.2	2.5	1.2	0.8	1.8	2.8		1.6	1.7	2.6	0.9	0.8	1.2	1.0	1.5	0.0
-248	1.3	1.4	1.5	0.9	1.1	2.6	0.8	0.3	2.6	2.6	1.3			2.2	0.0	0.7	0.6	1.5	1.8	0.0	1.1	1.7	2.0		0.5	1.4	2.9	0.6	0.5	1.3	0.0	0.6	0.0
-244	2.6	2.4	1.8	1.8	2.9	3.7	2.7	0.9	4.8	6.1	5.3			3.1	2.4	2.9	1.7	2.3	0.0	1.8	2.8	4.0	4.0		3.2	2.2	4.4	2.1	1.3	1.9	0.0	1.4	0.0
-230	1.3	0.7	0.8	1.4	0.8	2.3	1.1	1.2	1.7	1.4	0.0	1.1	0.9	1.0	1.8	1.3	1.8	1.6	1.2	1.2	1.5	2.3	1.6	0.9	0.9	1.4	2.1	1.6	2.3	1.8	1.2	2.5	1.2
-218	5.3	4.7	6.4	4.8	5.3		5.2	6.0	5.7	5.7	4.8	6.2	4.9	5.6	6.5	5.3	7.3	7.1	6.6	6.7	6.9	9.1	6.8	7.5	4.6	5.7	5.6	6.2	11.5	7.5	8.0	7.6	6.5
-205	6.8	5.1	5.6	6.2	8.3		7.3	5.6	7.1	4.7	3.9	7.1	6.8	7.4	9.4	6.4	10.5	9.8	7.6	8.2	8.1	13.2	8.8	7.8	5.9	8.9	8.7	6.3	15.3	9.5	6.1	11.9	8.3
-201	0.8	0.7	1.3	0.8	1.0		0.7	1.7	1.1	1.7	0.0	0.8	2.5	1.6	2.8	1.0	1.9	1.3	1.7	1.4	1.4	2.5	1.9	1.9	1.3	1.8	1.8	1.9	2.1	2.1	1.9	2.5	2.0
-198	0.9	0.9	0.0	0.6	0.0		0.7	0.6	1.4	0.9	0.0	0.8	1.0	0.8	0.9	0.8	1.0	1.1	0.7	1.5	0.7	1.1	0.9	1.2	0.9	1.1	0.9	1.0	0.0	1.8	0.0	0.9	0.0
-191	4.3	4.3	3.9	2.9	4.3	3.5	3.9	4.6	4.9	1.6	0.0	4.3	3.5	5.0	6.3	4.0	5.8	6.6	4.2	5.4	5.6	8.7	5.1	3.9	3.3	5.4	5.0	4.3	7.4	7.2	2.9	5.7	5.2
-189	0.7	1.3	1.0	0.9	1.5		1.4	1.4	1.7	1.0	0.0	1.1	1.1	1.7	1.8	1.0	1.8	1.8	1.2	1.4	1.5	2.5	1.9	2.1	1.5		1.5	1.5	2.3	1.7	0.0	1.8	0.0
-172	3.0	4.0	4.5	1.8	3.9	4.8	4.1	3.7	5.8	2.7	1.1	2.9	2.7	4.9	5.5	4.3	8.2	8.0		4.7	6.5		6.3	5.2	2.9	5.5	4.0	4.4	9.7	7.8	2.0	5.0	4.1
-167	2.3	2.4	3.6	2.2	2.5	1.9	0.9	2.6	3.8	1.7	0.0	2.5	2.2	2.3	4.0	2.1	4.5	4.0	2.3	2.2	4.1		4.2	2.1	2.1	3.1	1.8	2.3	5.3	4.2	0.0	4.4	0.0
-157	3.8	3.4	4.4	4.2	4.6	5.2	3.7	4.6	6.2	4.4	1.6	5.7	4.1	5.8	4.2	4.2	7.8	9.0		5.7	5.2	7.3	7.1	3.9	5.4	6.8	5.8	3.5	10.6	11.5	0.0	10.4	3.4
-149	0.9	2.0	1.5	0.8	1.7	1.8	1.4	2.0	2.0	1.6	1.0	1.5	1.3	1.1	1.3	1.2	2.9	3.0		1.5	1.5		2.0	1.3	1.5	1.1	2.2	1.1	1.9	2.7	0.0	1.8	0.0
-133	2.4	1.3	2.2	2.2	2.8	3.5	3.2	2.8	3.3	2.0	2.0	2.6	3.0	2.7	3.0	2.5	2.9	3.1		3.5	2.5		2.8	2.2	2.8	3.6	2.6	3.3	2.8	4.1	7.9	3.3	
-117			0.9	0.7	0.6	0.9	1.7	1.5	0.9	2.2	0.6	2.4	0.7	1.1	1.5	1.0	1.3	0.9	0.9	1.0	0.7	1.7	1.3	1.2	0.9	1.1	1.0	2.1	1.1	1.6	0.0	0.9	0.0
-64	3.1	2.9	1.3	22.5		7.9	1.7	2.3	2.8	3.7	2.5	6.2	2.6	3.1		1.2	1.7		3.2	1.6	2.5	3.4	2.7	4.3	6.3	5.0	3.1	2.0	1.9	3.8	2.1	2.2	1.3
-50	3.6	5.2	3.4	4.1		6.9	2.2		5.1	6.8	6.8	9.4	6.0	3.7		2.5	2.8		4.9	2.7	2.8		4.1	4.4	6.7	4.1	3.7	2.6	2.4	4.5	2.9	2.4	3.1
-18	3.8	4.9	4.1	3.6	3.7	3.6		3.1	4.1	4.0	4.8	9.6	4.8	4.0	4.4	3.6	3.8	2.9	3.1	3.3	3.6	3.0	3.2	4.3	3.4	3.9	2.7	3.5	3.9	3.7	3.8	3.8	4.1

Figure 6C Putative enhancer region; PBMCs

	Methylation (%)																															
Subject	1	2	3	4	5	6	7	8	9	10	11	12	13	14	15	16	17	18	19	20	21	22	23	24	25	26	27	28	29	30	31	32
CpG	Unstimulated cells																															
61820364	87.4	84.4	84.0	88.5	78.5	83.4	83.5	87.9	89.4	90.1	88.5		81.6	87.5	88.4	85.0	86.4	85.7	88.9	87.4	85.8	88.8	82.4	86.9	89.2	70.7	85.5	94.0	88.0	84.7	87.7	89.7
61820508	81.1	75.9	80.5	85.4	73.8	74.9	79.3	84.6	86.3	88.1	87.5		68.1	79.5	86.5	79.6	77.2	79.2	79.0	85.2	81.2	91.2		80.1	87.2	85.6	82.1	81.3	81.9	80.3	83.0	81.5
61820621	97.2	87.9	92.8	99.4	94.8	94.9	95.1	96.3	100	94.3	94.6		89.4	97.5	99.0	98.5	97.5	97.6	97.1	95.5	96.7	97.9	95.7	93.9	97.9	96.5	93.7	96.0	96.3	94.3	97.7	99.9
61820625	94.9	92.9	94.0	98.6	92.2	92.9	93.2	94.3	96.1	95.3	95.4		89.8	93.5	94.7	74.4	93.2	93.7	94.7	95.5	97.5	95.9	94.9	98.4	95.3		93.0	92.1	94.5	93.4	95.0	95.5
61820674	92.5	90.0	92.9	99.0	88.6	86.6	90.9	94.2	92.9	94.8	95.0		89.9	89.4	93.5	91.4	86.3	98.6	91.6	96.2	91.0	96.3	87.8	100	95.1	99.1	92.3	88.9	92.1	91.2	92.2	95.3
61820717	89.7	87.9	87.8	95.4	88.1	85.1	87.3	87.5	89.6	90.5	89.9		86.0	87.8	89.7	88.5	86.7	90.2	89.8	90.3	86.1	83.9	86.5	80.1		89.9	88.6	93.1	90.0	89.2	90.3	91.0
61820755	100.	100	96.8	93.8	94.1	89.8	94.2	93.0	98.2	95.4	95.2		88.1	100	93.3	95.1	100	100	90.2	100	96.1	95.0	92.2	100	100	100	94.9	93.0	94.0	95.3	96.7	100
61820814	94.1		92.4					95.0	93.5	100.					93.9	95.3			98.1		93.5	97.9	95.8	100	98.5			100	98.6	96.9	94.6	96.0
61820848	95.4	100	92.4					94.6	93.5	93.1					94.9	94.3			93.4		93.2	93.1	76.3		96.7				95.2	96.7	94.3	100
	Stimulated cells																															
61820364	82.5	81.4	87.3	81.2		81.9	81.5	89.9	78.3	89.1	82.1	84.2	85.7	89.3	83.6	85.1	85.4	88.1	85.0	86.1	89.0	80.0	86.3	88.2	87.1	83.5	86.0	90.1	86.2	82.2	89.7	88.9
61820508	71.0	75.4	84.9	69.9		75.4	81.6	82.6	84.0		73.0	68.7	75.6	84.7	79.8	74.3	77.2	79.8	85.0	79.8	90.9	67.7	77.2	84.3	84.5	79.2	78.5	88.6	70.9	79.2	86.0	80.3
61820621	89.7	92.5	94.9	93.1	93.8	92.2	96.4	99.3	94.0	98.6	91.0		93.3	96.7	95.8	92.6	91.0	92.3	90.4	91.0	95.1	95.7	86.3	95.3	96.1	92.9	95.1	94.2	96.0	91.4	100	97.6
61820625	92.2	92.8	92.7	91.4	94.6	90.1	94.0	97.2	94.4	74.2	88.0		92.9	94.4	92.5	92.6	92.6	95.0	93.5	96.4	94.2	97.5	98.9	93.3	94.7	94.0	92.9	95.0	94.1	88.1	95.7	96.0
61820674	85.6	90.6	94.0	90.3	90.1	88.6	90.9	93.1	93.4	99.5	92.2		90.4	94.5	89.5	89.0	87.0	88.4	91.5	90.5	98.8	100	92.9	93.0	91.5	91.2	92.6	91.5	91.3	100	94.0	94.2
61820717	87.0	86.4	90.1	86.6	89.2	86.3	89.2	79.1	89.8		89.5		87.7	88.1	90.2	83.5	88.6	89.3	89.1	88.6	89.4	94.3	94.5	89.9	88.9	88.5	87.9	87.1	85.8	92.7	90.0	90.8
61820755	90.1	90.2	93.1	91.3	89.5	89.7	100	100	100	100	100		93.5	92.2	92.8	100	90.8	91.6	93.1	100	96.9	97.1	100	94.8	93.8	91.7	94.1	92.4	92.8	86.7	92.7	96.8
61820814	98.5	93.2	92.0		93.0	82.6	93.5		99.5	93.9	94.4				95.9	99.8	96.2		93.8	95.0	93.7	91.7	91.2			97.1	93.7	99.7	99.2		99.1	
61820848	89.8	89.4	92.9		92.6		90.4		95.0	88.1	90.6				92.7	95.7	92.8		92.6	92.4	93.4	90.0	91.9			90.6	95.3	92.3	92.9		88.3	

Figure 7 *FADS2* DNA methylation in PBMCs and Jurkat cells

Figure 7A 5' Regulatory region; PBMCs

	Methylation (%)																																
Subject	1	2	3	4	5	6	7	8	9	10	11	12	13	14	15	16	17	18	19	20	21	22	23	24	25	26	27	28	29	30	31	32	33
CpG																																	
-1661	94.4	94.5	95.2	94.6	94.6	94.6	95.0	95.5	96.5	96.2	95.2	95.0	96.7	95.6	95.8	96.5	94.7	94.7	95.1	96.1	95.8	94.6	93.9	95.1	95.3	94.2	93.5	94.6	95.8	94.6	96.0	95.8	96.4
-1655	83.6	87.1	85.1	86.2	87.2	89.8	89.4	88.0	89.6	88.3	88.0	87.5	87.4	86.8	86.5	87.0	87.6	88.1	88.9	89.7	87.8	83.4	85.1	86.6	88.2	82.9	77.9	87.0	88.2	85.5	87.1	86.7	87.8
-1337	77.1	76.1	77.9	73.0	74.6	80.7	81.4	76.2	78.8	82.4	83.0	82.0	84.2	78.3	81.7	78.7	78.7	80.7	80.8	80.0	81.8	74.6	75.8	82.1	81.9	77.3	74.0	76.1	80.1	73.8	75.4	82.0	79.3
-1278	62.1	68.6	59.6	63.4	66.5	73.0	72.4	60.9	64.8	65.6	64.1	70.3	68.0	65.5	67.9	72.4	71.6	69.1	71.5	63.4	68.6	51.2	64.4	69.1	59.8	52.3	55.9	70.7	72.1	68.9	71.9	64.1	65.0
-1156	58.8	58.7	57.6	50.0	50.0	62.9	59.1	49.7	52.6	55.4	56.6	65.1	71.0	61.8	51.7	68.9	58.8	53.8	57.4	53.1	62.9	39.0	49.9	58.9	47.0	50.3	43.1	55.5	59.1	53.8	55.9	54.6	53.7
-1119	24.0	24.2	20.7	17.1	21.3	27.6	30.3	17.2	17.7	13.8	16.5	23.5	28.1	19.5	15.0	19.4	24.6	14.6	22.2	20.2	22.5	10.2	23.4	23.0	13.7	19.8	16.8	16.1	20.6	18.1	15.5	22.1	
-1112	37.2	33.6	32.9	27.8	29.6	37.8	38.4	27.3	27.6	24.8	25.0	38.2	41.3	33.8	27.6	32.0	37.7	25.3	32.5	29.1	38.0	15.9	32.4	24.9	23.0	28.0	28.2	28.4	31.0	29.1	34.6	30.8	
-1101	31.5	34.0	27.5	25.0	29.6	36.6	36.1	25.5	25.1	20.4	21.7	37.2	37.7	32.0	28.2	30.3	34.5	24.2	27.1	26.6	31.6	17.0	34.0	29.4	19.4	25.5	25.4	24.6	29.3	25.6	27.8	26.4	
-1071	45.8	47.8	39.0	40.0	41.2	52.5	49.8	37.5	43.4	38.4	43.6	54.4	56.1	50.9		48.8	52.1	41.8	50.5	46.9	53.7	26.5	44.6	51.7	42.8	41.6	37.9		47.4		43.8	46.8	
-1067	39.1	37.5	36.6	28.5	33.4	41.2	45.0	31.7	33.5	28.5	26.0	44.3	41.2	35.8		41.0	39.4	33.6	37.1	29.7	5.5	17.2	36.0	41.6	27.4	30.1	29.8		35.1		37.7	34.5	
-1056	50.0	48.1	46.0	42.3	42.8	52.2	52.8	39.4	51.0	40.6	37.7	52.8	57.8	47.5		45.5	55.5	44.1	48.6	51.5	60.7	27.3	48.3	52.6	39.6	1.5	37.4		47.2		49.0	47.1	
-1013	57.5	62.9	61.5	57.9	30.3	72.2	67.0	57.2	66.8	55.2	65.1		60.9	63.1	55.6	63.1	65.9	62.6	69.0	63.8	73.7	48.8	60.8	65.2	56.6	52.6	53.0	61.6	72.3	67.6	61.6	60.7	62.2
-980	41.6	43.2	39.0	36.6	20.9	48.7	47.5	34.8	36.6	28.8	32.9		42.5	40.5	32.1	36.9	39.9	35.0	40.8	35.0	43.0	21.0	43.4	41.8	27.4	31.2	32.2	39.1	40.7	36.5	40.7	37.1	39.0
-975	47.5	54.4	46.0	45.9	24.1	62.7	56.2	41.3	51.2	39.1	48.2		50.6	56.8	41.2	50.7	54.3	46.7	55.4	48.8	58.9	31.9	48.3	57.3	41.1	39.9	43.7	51.5	60.7	44.8	52.5	53.4	51.0
-914	19.5	18.6	14.6		15.8	21.6	25.2	13.8	12.9	14.3	12.9	20.8	20.9	16.0	14.9	16.2	15.5	13.9			16.1	5.5	17.1	16.4	11.0	0.7	10.3	12.0	15.6	14.3		14.9	
-871	26.2	24.0	19.0	16.7	24.0	23.3	26.8	13.7	14.5	14.3	12.1	29.1	32.1	20.8	19.7	19.2	23.4	19.8	22.7	13.8	22.4		28.1	23.8	10.7	14.3	14.8	18.0	17.3	20.3	20.1	17.2	20.6
-869	26.5	24.8	16.0	15.2	21.1	27.1	27.5	13.0	16.2	12.6	12.3	29.6	32.2	18.6	17.4	21.2	25.1	19.9	31.2	14.5	22.1		28.8	24.0	0.8	11.6	17.1	18.6	22.3	1.3	21.4	21.8	18.0
-855	28.0	25.9	21.3	18.4	23.1	25.6	29.6	15.3	18.5	18.0	14.7	33.6	32.1	24.6	17.0	21.5	24.2	18.7		17.1	23.3		11.0	24.9	15.1		18.8	18.3	22.2	21.6	22.0	22.9	19.3
-817	22.4	23.2	19.5	15.4	19.0	22.2	21.2	12.4	12.1	11.5	10.7	23.6	27.4	15.1	14.6	18.9	22.1	13.5	18.1	14.3	17.6		23.5	34.5	23.2	27.2	34.7	16.6	17.2	17.1	16.6	14.6	17.6
-806	28.8	28.6	19.2	18.9	21.4	29.8	27.3	14.3	16.8	13.4	12.5	25.3	30.8	25.8	18.7	24.7	28.7	19.4	24.0	16.7	22.6		25.5	20.3	13.9	16.9	15.8	19.0	21.8	20.7	21.6	22.4	21.1
-775	29.3	30.1	21.7	24.2		40.1		17.2	19.2		13.8	38.8	34.3	27.2	24.1	30.3			29.3		29.3		30.2	31.8	29.8	19.5	17.6	24.1	24.3	26.6	25.8	28.5	
-718	4.5	9.3	9.7	6.5	9.7	9.7	9.8	8.1	6.6	8.5	4.8	10.4	13.0	8.1	8.0	6.3	10.0	7.3	9.9	4.4	13.6		11.2	10.7	5.6	5.3	7.5	6.3		9.8	6.2	7.6	
-686	11.5	9.9	7.2	7.5	10.4	9.5	12.4	5.8	6.1	5.1	4.3	12.7	11.9	7.3	7.3	9.9	9.0	7.7	8.0	6.3	9.3		12.0	10.6	5.3	6.4	6.5	7.2	6.8	8.5	8.4	7.1	7.6
-669	18.9	16.1	17.1	12.9	14.6	14.4	18.5	11.0	14.4	15.8	13.9			17.3	12.8	13.9	15.1	11.0	19.1	13.1	17.0		19.3	16.7	13.7	13.0	13.0	15.1	11.5	10.9	13.8		12.4
-667		15.1		12.8	11.7	15.3	14.0	10.5	6.2	9.1	5.6		15.1	10.8	9.5	12.6	13.7	11.2	11.4	7.6	19.0						11.1	10.1	10.9	11.5	14.0	14.1	9.0
-374	1.5	0.0	1.3	0.7	1.8	1.6	1.7	2.4	1.4	2.1	0.0		0.0	1.5	1.9	0.0	0.0	0.0	1.0	2.1	1.5	0.0	1.1	0.0	1.8	0.0	0.8	0.7	0.0	0.0	0.0	1.6	2.0
-368	1.7	1.1	0.7	1.0	1.7	1.6	0.0	1.9	2.7	0.9	1.2	0.0	0.0	2.8	1.6	1.7	1.6	0.0	0.9	2.2	0.8	0.0	0.9	0.9	1.0	2.3	1.0	2.8	0.0	0.0	1.8	1.3	0.8
-356	7.3	7.6	7.3	3.6	7.8	8.8	4.4	11.8	12.2	7.5	10.1	0.0	4.7	8.3	9.2	11.4	5.5	6.9	5.9	7.5	8.4	6.0	11.3	6.6	6.9	4.8	6.4	7.7	7.7	5.9	5.3	7.6	7.5
-344	3.8	2.4	3.6	3.1	3.8	5.5	4.2	8.1	6.5	3.4	4.1	0.0	1.9	3.4	5.0	7.0	5.1	3.3	2.9	6.7	5.9	6.1	3.6	4.0	3.8	3.1	4.5	3.4	3.9	3.2	3.3	4.7	4.4
-336	3.0	2.3	2.9	1.9	2.3	2.3	1.0	4.7	4.9	2.4	4.3	0.0	1.6	3.8	3.3	4.0	3.2	2.6	1.0	4.0	2.8	2.2	2.9	2.3	2.5	1.3	2.0	1.6	3.0	2.7	1.5	4.2	1.9
-334	3.5	0.0	2.0	4.0	2.1	4.0	2.6	5.4	1.6	2.9	3.0		1.3	2.1	3.5	2.2	0.0	0.0	2.9	3.1	4.8	0.0	2.6	0.0	1.7	2.7	1.1	0.0	2.2	2.4	2.6	3.0	0.0
-258	0.8	0.0	1.8	0.9	0.7	0.9	0.7	1.1	0.8	1.4	0.9	1.1	0.6	1.0		1.0	1.2	0.0	0.8	0.0	1.0	0.0	1.7	0.8	1.2	0.7		3.0				1.1	1.9
-253	1.6	2.6	2.7	1.7	2.3	2.6	1.2	1.7	1.0	2.2	0.9	1.5	3.1	1.9		1.6	1.2	0.0	1.9	1.1	1.7	0.0	1.4	1.3	1.3	0.8	1.6	2.5	0.0			2.1	2.1
-250	0.7	0.9	2.5	1.0	1.0	1.0	0.9	1.1	0.8	1.2	0.7	0.8	1.1	1.2	0.0	1.1	1.2	0.0	0.8	0.0	0.6	0.0	1.1	0.9	1.0	0.9	2.4	3.6	1.3		0.0	1.1	4.4
-248	0.9	1.2	2.0	1.0	0.8	1.0	0.7	0.6	0.5	1.3	0.6	1.3	1.1	0.9	1.5	1.3	0.8	0.0	0.7	0.0	0.5	0.0	1.0	0.6	0.7	0.8	2.2	3.5	0.7		0.0	0.7	
-244	1.8	1.8	4.1	2.1	1.9	2.6	1.5	1.7	1.8	2.0	1.2	1.8	2.1	2.4		1.4	1.6	0.0	1.8	0.0	1.6	0.0	2.5	1.8	1.1	2.0	3.4	5.6	1.5		0.0	1.9	
-230	1.2	0.8	0.9	0.9	0.8	1.6	0.9	2.3	2.7	1.7	1.3	0.8	0.5	1.3	1.5	1.7	1.6	0.7	0.9	2.4	1.4	0.0	1.2	1.4	1.0	1.6	0.9	1.1	1.1	2.0	0.8	1.9	1.0
-218	5.5	5.1	7.1	6.4	4.4	6.4	5.3	8.2	8.8	6.0	6.8	4.3	4.6	5.4	6.6	6.6	4.0	6.1	5.0	9.2	8.0	4.5	7.0	5.7	5.3	6.0	5.1	5.6	5.2	5.9	5.1	5.6	7.2
-205	7.6	7.4	7.2	6.3	3.8	9.5	6.6	12.0	13.2	6.8	10.0	3.2	3.6		8.4	8.4	6.4	6.8	7.3	12.6	10.2	6.5	8.5	6.8	6.9	7.1	5.3	6.4	7.0	7.3	6.4	9.5	7.3
-201	1.9	1.6	1.0	1.1	0.5	1.9	1.6	3.1	2.2	1.6	1.8	0.9	0.7		1.6	2.1	1.2	1.3	1.9	3.4	2.8	1.4	1.8	1.5	1.8	1.5	1.1	1.7	1.4	2.2	1.5	2.0	1.4
-198	1.0	0.0	1.2	0.7	0.7	1.4	1.1	1.0	1.3	1.0	0.0	0.0	0.7		1.0	0.0	1.1	0.0	0.6	0.0	1.0	0.0	0.7	0.7	1.0	0.7	0.4	0.8	0.0	0.9	0.0	1.5	1.0
-191	5.1	4.9	3.5	5.3	1.4	7.0	4.2	8.0	8.4	4.7	6.5	0.0	1.4		6.3	5.6	3.5		3.6	7.0	7.1	3.8	5.5	4.3		5.5	2.8	4.7		4.4	4.5	6.8	5.5
-189	1.6	0.0	1.8	1.4	1.0	2.2	1.5	2.9	2.6	1.6	1.6	0.0	0.0		1.5	1.2	1.8		1.1	1.3	0.0	0.0	1.9	1.5	1.0	1.5	0.8	1.0		1.8	1.0	1.5	0.9
-172	4.6	4.6	4.7	4.0	1.4	6.5	3.4	9.1	9.5	3.8	5.8	1.4	1.1	4.2	5.3	5.8	3.6	2.8	3.0	7.5	6.5	2.6	5.2	4.0	4.4	5.0	4.1	3.2	3.8	3.6	2.8	8.2	4.5
-167	3.6	1.0	2.6	2.6	0.7	4.7	2.3	5.6	5.5	2.5	4.3	0.0	1.5	2.3	3.6	1.8	2.2	0.8	2.4	3.8	0.0	0.9	2.7	2.4	2.5	0.0	2.1	1.7	1.7	2.8	1.3	3.0	1.5
-157	5.5	4.4	4.8	5.1	2.1	8.8	3.9	10.7	10.3	3.9	6.7	1.5	1.6	4.4	6.3	5.4	4.0	3.9	3.5	8.9	7.7	3.2	4.9	5.1	4.5	4.4	3.8	3.8	4.4	4.1	4.1	7.0	5.1
-149	1.6	0.0	1.2	1.0	0.7	2.5	1.0	3.5	3.0	1.3	0.9	0.0	0.7	0.9	2.2	0.9	1.2	0.0	1.4	1.5	1.5	0.0	2.1	1.1	1.4	1.5	1.4	1.6	0.9	1.8	0.6	2.2	1.3
-133	2.7	2.1	2.5	2.4	1.8	3.0	2.4	3.3	3.5	2.6	3.0	2.1	1.5	2.1	3.2	3.0	3.4	2.0	2.6	3.0	3.5	2.0	3.5	2.1	2.3	2.8	2.6	2.2	2.4	2.6	2.2	2.5	1.9
-117	1.2	0.0	2.4	0.9	1.5	1.2	1.1	1.9	1.4	1.4	0.8	0.9	1.2	0.8	1.3	0.0	1.7	0.0	0.9	0.0	1.3	0.0	1.3	0.9	1.4	0.6	1.4	1.2	0.9	1.3	0.5	1.2	1.3
-64	1.8	1.4		1.5	1.9	1.7	1.7	1.9	1.6	2.0	1.4	2.2	1.7	2.1	7.2		1.9	1.4	1.9	1.2	2.2	1.2	1.9	1.5	1.8	1.1	4.2	2.4	1.8	2.2		2.1	2.7
-50	2.8	2.5		2.4	2.8	2.9	2.8	2.3	2.6	2.2	1.6	2.8	3.2	2.8	4.1		2.4	2.7	2.5	2.9	2.7	2.5	3.1	2.5	2.4	8.6	4.6	3.6	6.0	4.3		2.5	6.6
-18	3.3	3.6	4.9	3.3	3.7	3.8	3.8	3.6	2.8	3.3	2.1	3.0	3.8	4.0	3.2	3.1	4.0	3.8	3.2	3.5	3.3	2.8	3.6	3.7	3.4	2.9	4.6	6.1	3.3	3.1	3.4	3.3	3.5

Figure 7A 5’ Regulatory region; Jurkat cells

	Methylation (%)									
Replicate	1	2	3	4	5	6	7	8	9	10
CpG										
-1661	95.5	94.8	95.4	95.6	96.4	95.1	94.7	94.9	95.0	92.3
-1655	85.2	87.0	86.3	86.2	84.6	85.3	85.1	86.0	89.2	80.7
-1337	57.3	57.0	52.1	60.0	57.7	54.1	56.9	57.5	50.9	55.1
-1278	59.3	57.3	56.5	51.3	57.3	54.6	59.6	66.0	67.2	73.1
-1156	24.0	24.6	21.1	24.5	25.9	24.9	24.6	23.1	24.1	24.5
-1119	7.4	6.2		7.4	6.2	7.2	6.3	6.1	7.6	8.0
-1112	13.2	13.3	15.0	12.2	14.4	13.0	12.7	13.9	13.0	13.1
-1101	20.8	23.1	22.7	25.3	25.9	22.3	22.1	19.4	20.1	19.9
-1071	24.4	24.2	21.6	21.4	24.5	23.5	19.8	19.8	22.5	22.8
-1067	23.6	22.5	22.0	21.6	25.4	24.1	6.6	20.6	23.1	23.2
-1056	32.2	35.7	32.5	31.5	34.4	35.6	31.0	31.7	30.9	33.5
-1013	19.1	19.4	18.2	17.6	18.1	18.6	18.5	18.3	19.4	18.5
-980	18.6	17.8	18.2	17.8	18.1	19.9	17.7	17.0	18.6	18.3
-975	17.1	17.2	16.4	17.2	17.7	17.9	15.9	16.8	19.1	17.2
-914	3.2	3.3	4.5	4.1			0.4	4.3	5.5	2.9
-871	5.0	4.0	5.8	4.2	3.3	6.5	4.8	5.6	5.6	3.3
-869	2.9	4.2	4.0	3.8	3.5	5.4	3.4	3.7	5.6	2.9
-855	4.8	6.3	5.0	6.3	6.5	7.3	7.2	4.3	6.7	5.5
-817	4.3	4.9		18.5	7.0	6.7	5.0	5.2	4.4	13.6
-806	2.9		4.7	1.3	6.3	5.5	4.0	9.4		3.9
-775		2.9	2.6		7.1	3.7	4.4	2.4	3.2	5.9
-718	2.0	3.2	1.5	2.1	3.9	2.9	3.0	2.9	2.6	
-686	0.9	1.0	1.2	0.7	1.0	0.7	0.9	1.6	1.1	
-669	6.7	9.7	6.8	8.1		9.4	7.5	7.3	11.4	6.7
-667	0.0	0.0	0.0	0.0	0.0	0.0	0.0	0.0	0.0	0.0
-374	1.4	1.0	0.0	1.7	0.0	0.6	1.0	1.7	2.1	0.9
-368	1.4	1.7	0.0	0.8	1.0	1.4	1.0	1.2	1.7	1.3
-356	7.3	7.0	6.7	6.8	6.0	7.8	6.6	5.5	6.5	6.0
-344	2.6	5.2	3.2	2.8	3.4	4.4	3.6	3.8	4.4	4.3
-336	4.0	2.7	2.8	2.0	2.8	1.5	1.4	2.7	2.2	3.3
-334	2.0	1.2	1.5	0.0	1.9	0.0	1.8	2.5	2.7	2.2
-258	0.9	0.0	1.6	0.0	0.9	0.0	1.0	3.2	1.0	1.5
-253	1.2	2.0	1.2	2.0	2.6		1.7	1.7	1.6	1.5
-250	1.0	1.0	1.4	0.8	0.8	1.4	0.0	1.2	3.1	1.2
-248	0.0	0.9	0.0	0.5	0.5	1.0	0.0	0.7	0.0	0.8
-244	1.0	1.7	0.0	1.4	0.9	1.6	0.0	2.1	2.4	1.7
-230	1.3	1.6	0.6	1.0	1.1	1.1	1.0	0.9	1.0	1.2
-218	6.0	6.4	4.8	4.9	5.8	4.7	5.5	5.8	6.1	4.8
-205	7.3	6.5	6.2	6.7	6.8	6.1	6.7	5.8	6.5	6.7
-201	2.1	1.1	1.0	1.1	1.2	1.1	0.0	1.0	1.4	1.7
-198	0.6	0.8	0.0	0.6	0.0	0.8	0.0	0.6	1.0	0.7
-191	6.1	6.1	4.0	4.4	3.8	4.3	4.6	4.6	3.9	4.4
-189	0.8	1.1	0.0	1.0	0.0	1.3	0.0	1.0	1.2	1.2
-172	4.4	4.4	2.7	4.0	3.6	4.2	3.7	4.1	4.3	3.9
-167	2.6	2.9	0.0	2.7	0.0	2.1	0.8	2.3	2.7	1.8
-157	5.0	7.0	3.4	4.6	4.3	4.6	4.4	10.0	5.0	4.8
-149	2.3	1.5	0.0	1.1	0.0	1.3	0.0	1.3	1.2	0.9
-133	2.7	2.9	2.0	8.6	2.1	2.3	2.2	2.4	2.6	1.9
-117	0.0	1.4	0.0	0.9		1.4	0.0	1.0	0.9	0.6
-64	1.4	2.0	1.8		1.3	1.8		2.1	2.0	2.0
-50	1.7	2.4	2.4		2.2	2.0		2.6	2.4	3.1
-18	3.1	3.2	2.9	4.0	3.4	3.0	3.5	3.6	2.9	3.0

Figure 7C Putative enhancer region; unstimulated PBMCs and Jurkat cells

	Methylation (%)																															
Subject	1	2	3	4	5	6	7	8	9	10	11	12	13	14	15	16	17	18	19	20	21	22	23	24	25	26	27	28	29	30	31	32
CpG	Unstimulated PBMCs																															
61820364	87.4	84.4	84.0	88.5	78.5	83.4	83.5	87.9	89.4	90.1	88.5		81.6	87.5	88.4	85.0	86.4	85.7	88.9	87.4	85.8	88.8	82.4	86.9	89.2	70.7	85.5	94.0	88.0	84.7	87.7	89.7
61820508	81.1	75.9	80.5	85.4	73.8	74.9	79.3	84.6	86.3	88.1	87.5		68.1	79.5	86.5	79.6	77.2	79.2	79.0	85.2	81.2	91.2		80.1	87.2	85.6	82.1	81.3	81.9	80.3	83.0	81.5
61820621	97.2	87.9	92.8	99.4	94.8	94.9	95.1	96.3	100	94.3	94.6		89.4	97.5	99.0	98.5	97.5	97.6	97.1	95.5	96.7	97.9	95.7	93.9	97.9	96.5	93.7	96.0	96.3	94.3	97.7	99.9
61820625	94.9	92.9	94.0	98.6	92.2	92.9	93.2	94.3	96.1	95.3	95.4		89.8	93.5	94.7	74.4	93.2	93.7	94.7	95.5	97.5	95.9	94.9	98.4	95.3		93.0	92.1	94.5	93.4	95.0	95.5
61820674	92.5	90.0	92.9	99.0	88.6	86.6	90.9	94.2	92.9	94.8	95.0		89.9	89.4	93.5	91.4	86.3	98.6	91.6	96.2	91.0	96.3	87.8	100	95.1	99.1	92.3	88.9	92.1	91.2	92.2	95.3
61820717	89.7	87.9	87.8	95.4	88.1	85.1	87.3	87.5	89.6	90.5	89.9		86.0	87.8	89.7	88.5	86.7	90.2	89.8	90.3	86.1	83.9	86.5	80.1		89.9	88.6	93.1	90.0	89.2	90.3	91.0
61820755	100.	100	96.8	93.8	94.1	89.8	94.2	93.0	98.2	95.4	95.2		88.1	100	93.3	95.1	100	100	90.2	100	96.1	95.0	92.2	100	100	100	94.9	93.0	94.0	95.3	96.7	100
61820814	94.1		92.4					95.0	93.5	100.					93.9	95.3			98.1		93.5	97.9	95.8	100	98.5			100	98.6	96.9	94.6	96.0
61820848	95.4	100	92.4					94.6	93.5	93.1					94.9	94.3			93.4		93.2	93.1	76.3		96.7				95.2	96.7	94.3	100
	Jurkat cells																															
61820364	90.5	90.8	90.1	91.2	90.4	90.2	90.7	91.0	85.2	90.6	90.5																					
61820508	83.9	83.6	83.5	83.8	84.4	83.8	84.5	83.6	73.8	84.2	83.9																					
61820621	97.0	97.5	96.5	98.0	94.6	95.0	96.8	95.6	95.0	100	97.0																					
61820625	94.6	96.4	94.9	94.0	94.7	94.4	94.9	94.8	92.4	99.0	94.6																					
61820674	91.8	92.5	98.7	91.6	98.9	93.1	92.5	98.3	94.4	100	91.8																					
61820717	64.6	83.0	66.5	63.7	64.7	63.7	62.8	61.8	62.3	91.8	64.6																					
61820755	92.2	90.5	94.2		93.3			91.3	98.6	90.6	92.2																					
61820814	97.4	94.6	97.3	93.8		92.4				92.1	97.4																					
61820848	94.9	91.6	91.5	91.1		92.0			96.7	92.7	94.9																					
-18	90.5	90.8	90.1	91.2	90.4	90.2	90.7	91.0	85.2	90.6	90.5																					
